# Supplementary material for: Strategies to assess and promote the socio-emotional competencies of university students in the socio-educational and healthcare fields: A scoping review
Source: PLoS One. 2025 May 22;20(5):e0324531. doi: 10.1371/journal.pone.0324531 (PMC12097715; doi:10.1371/journal.pone.0324531)
Supplement: S1 Table — (DOCX) [file pone.0324531.s001.docx]

**S1 Table.** Main theoretical frameworks of SECs.

| **Theoretical frameworks** | | **Socio-emotional competencies** |
| --- | --- | --- |
| **Emotional Intelligence** | Petrides & Furnham 2001  [1] | - Well-being - Self-control - Emotionality - Sociability |
|  | Salovey & Mayer 1990  [2] | - Perceiving emotions - Facilitating though using emotion - Understanding emotions - Managing emotions |
|  | Bar- On 2006  [3] | - Intrapersonal skills - Interpersonal skills - Stress management - Adaptation skills - General mood |
|  | Goleman 1995  [4] | - Self-awareness - Self-management - Social awareness - Relationship management |
|  | Mikolajczak 2010  [5] | - Knowledge: the complexity and width of emotion knowledge, the beliefs about emotions. - Abilities: the ability to apply knowledge to a problem-solving situation and to implement a given strategy. - Disposition: the propensity to put one’s abilities into practice, the frequency with which one uses his/her abilities. |
| **Social and Emotional Learning** | Casel 2016  [6] | - Self-awareness - Self-management - Social awareness - Relationship skills - Responsible decision-making |
| **Emotional Education** | Bisquerra 2003  [7] | - Emotional awareness - Emotional regulation - Emotional autonomy - Social competence - Competencies for life and well-being |

*Notes*.

1. Petrides K V., Furnham A. Trait emotional intelligence: Psychometric investigation with reference to established trait taxonomies. Eur J Pers. 2001;15: 425–448. doi:https://doi.org/10.1002/per.416

2. Salovey P, Mayer JD. Emotional intelligence. Imagin Cogn Pers. 1990;9: 185–211. doi:10.2190/DUGG-P24E-52WK-6CDG

3. Bar-On R. The Bar-On model of emotional-social intelligence (ESI). Psicothema. 2006;18: 13–25.

4. Goleman D. Emotional intelligence: Why it can matter more than IQ for character, health and lifelong achievement. Bantam Books. New York; 1995.

5. Mikolajczak M. Going Beyond The Ability-Trait Debate: The Three-Level Model of Emotional Intelligence. E-Journal Appl Psychol. 2010;5: 25–31.

6. Collaborative for Academic Social and Emotional Learning. Marco de SEL de CASEL: ¿Cuáles son las áreas de competencias principales y dónde se promueven? 2020. pp. 1–4. Available: https://casel.org/sel-framework/

7. Bisquerra R. Educación emocional y competencias básicas para la vida. Rev Investig Educ. 2003;21: 7–43.
