# Supplementary material for: Strategies to assess and promote the socio-emotional competencies of university students in the socio-educational and healthcare fields: A scoping review
Source: PLoS One. 2025 May 22;20(5):e0324531. doi: 10.1371/journal.pone.0324531 (PMC12097715; doi:10.1371/journal.pone.0324531)
Supplement: S2 Table — (DOCX) [file pone.0324531.s002.docx]

**S2 Table.** Search strategy of each database

**a.** Scopus search strategy

| Step | Search terms |
| --- | --- |
| #1 | Search within: TITLE-ABS-KEY  ( "emotional skill*" OR "socioemotional skill*" OR "socio-emotional skill*" OR "emotional competenc*" OR "socioemotional competenc*" OR "socio-emotional competenc*" OR "emotional abilit*" OR "socioemotional abilit*" OR "socio-emotional abilit*" OR "emotional intelligence" OR "socioemotional intelligence" OR "socio-emotional intelligence" OR "emotional education" OR "socioemotional education" OR "socio-emotional education" OR "emotional learning" OR "socioemotional learning" OR "socio-emotional learning" ) ) |
| #2 | Search within: TITLE-ABS-KEY  ( undergraduat* OR postgraduat* OR graduat* OR universit* OR postsecondary OR college OR "higher education" OR "tertiary education" OR bachelor* OR professionalism ) |
| #3 | Search within: TITLE-ABS-KEY  ( TITLE-ABS-KEY ( addiction OR substance OR abuse OR dependen* OR alcohol OR binge OR smoke OR gambl* OR deaf* OR posttraum* OR trauma* OR depressi* OR disorder OR bulimic OR hyperactivity OR suicid* OR psychiatric* OR patholog* OR syndrome OR autis* OR schizophreni* OR psychopath* OR bipolar OR self-injur* OR self-harm OR harassment OR stalking OR cyberbullying OR diagnostic* OR agressi* OR violence OR maltreatment OR sex* OR spiritual* OR victim* OR child* OR parent* OR couple OR cultural OR insomnia OR stress OR distress OR anxiety OR aggress* ) |
| #4 | (#1 AND #2) AND NOT #3 |
| #5 | FILTER: Publication Year (2000-2024) |
| #6 | FILTER: ( LIMIT-TO ( DOCTYPE , "ar" ) OR LIMIT-TO ( DOCTYPE , "sh" ) |
| #7 | FILTER: ( PUBSTAGE , "final" ) |
| #8 | FILTER: ( LIMIT-TO ( SUBJAREA , "soci" ) OR LIMIT-TO ( SUBJAREA , "medi" ) OR LIMIT-TO ( SUBJAREA , "psyc" ) OR LIMIT-TO ( SUBJAREA , "nurs" ) OR LIMIT-TO ( SUBJAREA , "heal" ) OR LIMIT-TO ( SUBJAREA , "mult" ) OR LIMIT-TO ( SUBJAREA , "dent" ) |
| #9 | FILTER: ( LIMIT-TO ( LANGUAGE , "english" ) OR LIMIT-TO ( LANGUAGE , "spanish" ) |
| #10 | ALERTS ACTIVATED: Yes |

**b.** PsycINFO search strategy

| Step | Search terms |
| --- | --- |
| #1 | ((((MJMAINSUBJECT.EXACT("Emotional Assessment") OR MJMAINSUBJECT.EXACT("Self-Knowledge") OR MJMAINSUBJECT.EXACT("Emotional Regulation") OR MJMAINSUBJECT.EXACT("Emotional Intelligence") OR MJMAINSUBJECT.EXACT("Self-Control") OR MJMAINSUBJECT.EXACT("Expressed Emotion") OR MJMAINSUBJECT.EXACT("Social Emotional Learning") OR MJMAINSUBJECT.EXACT("Emotion Recognition") OR MJMAINSUBJECT.EXACT("Emotional Control")) OR tiab("emotional skill*" OR "socioemotional skill*" OR "socio-emotional skill*" OR "emotional competenc*" OR "socioemotional competenc*" OR "socio-emotional competenc*" OR "emotional abilit*" OR "socioemotional abilit*" OR "socio-emotional abilit*" OR "emotional intelligence" OR "socioemotional intelligence" OR "socio-emotional intelligence" OR "emotional education" OR "socioemotional education" OR "socio-emotional education" OR "emotional learning" OR "socioemotional learning" OR "socio-emotional learning" OR "emotional assess*" OR "self-knowledge" OR "emotional regulation" OR "self-control" OR "expressed emotion*" OR "emotion recognition" OR "emotional control") OR if("emotional skill*" OR "socioemotional skill*" OR "socio-emotional skill*" OR "emotional competenc*" OR "socioemotional competenc*" OR "socio-emotional competenc*" OR "emotional abilit*" OR "socioemotional abilit*" OR "socio-emotional abilit*" OR "emotional intelligence" OR "socioemotional intelligence" OR "socio-emotional intelligence" OR "emotional education" OR "socioemotional education" OR "socio-emotional education" OR "emotional learning" OR "socioemotional learning" OR "socio-emotional learning" OR "emotional assess*" OR "self-knowledge" OR "emotional regulation" OR "self-control" OR "expressed emotion*" OR "emotion recognition" OR "emotional control")) |
| #2 | ((MJMAINSUBJECT.EXACT("Colleges") OR MJMAINSUBJECT.EXACT("Professionalism") OR MJMAINSUBJECT.EXACT("College Graduates") OR MJMAINSUBJECT.EXACT("Graduate Students") OR MJMAINSUBJECT.EXACT("College Students") OR MJMAINSUBJECT.EXACT("Postgraduate Students") OR MJMAINSUBJECT.EXACT("Postgraduate Training") OR MJMAINSUBJECT.EXACT("Undergraduate Education") OR MJMAINSUBJECT.EXACT("Graduate Education") OR MJMAINSUBJECT.EXACT("Higher Education") OR MJMAINSUBJECT.EXACT("Educational Degrees")) OR tiab(Undergraduat* OR Postgraduat* OR graduat* OR universit* OR postsecondary OR college OR "higher education" OR "tertiary education" OR bachelor* OR professionalism OR "educational degree*") OR if(Undergraduat* OR Postgraduat* OR graduat* OR universit* OR postsecondary OR college OR "higher education" OR "tertiary education" OR bachelor* OR professionalism OR "educational degree*"))) |
| #3 | (tiab(addiction OR substance OR abuse OR dependen* OR alcohol OR binge OR smoke OR gambl* OR deaf* OR posttraum* OR trauma* OR depressi* OR disorder OR bulimic OR hyperactivity OR suicid* OR psychiatric* OR patholog* OR syndrome OR autis* OR schizophreni* OR psychopath* OR bipolar OR self-injur* OR self-harm OR harassment OR stalking OR cyberbullying OR diagnostic* OR agressi* OR violence OR maltreatment OR sex* OR spiritual* OR victim* OR child* OR parent* OR couple OR cultural OR insomnia OR stress OR distress OR anxiety OR aggress*) OR if(addiction OR substance OR abuse OR dependen* OR alcohol OR binge OR smoke OR gambl* OR deaf* OR posttraum* OR trauma* OR depressi* OR disorder OR bulimic OR hyperactivity OR suicid* OR psychiatric* OR patholog* OR syndrome OR autis* OR schizophreni* OR psychopath* OR bipolar OR self-injur* OR self-harm OR harassment OR stalking OR cyberbullying OR diagnostic* OR agressi* OR violence OR maltreatment OR sex* OR spiritual* OR victim* OR child* OR parent* OR couple OR cultural OR insomnia OR stress OR distress OR anxiety OR aggress*))) |
| #4 | (#1 AND #2) NOT #3 |
| #5 | FILTER: Publication Year (2000-2024) |
| #6 | FILTER: PEER(yes) |
| #7 | FILTER: subject.exact("Adulthood (18 yrs & older)") |
| #8 | FILTER: me.exact("Empirical Study" OR "Quantitative Study" OR "Interview" OR "Qualitative Study" OR "Longitudinal Study" OR "Prospective Study" OR "Experimental Replication" OR "Focus Group" OR "Followup Study" OR "Retrospective Study" OR "Field Study" OR "Nonclinical Case Study") |
| #9 | FILTER: (la.exact("ENG" OR "SPA") |
| #10 | ALERTS ACTIVATED: Yes |

**c.** PubMed search strategy

| Step | Search terms |
| --- | --- |
| #1 | ((("emotional skill*"[Title/Abstract] OR "socioemotional skill*"[Title/Abstract] OR "socio emotional skill*"[Title/Abstract] OR "emotional competenc*"[Title/Abstract] OR "socioemotional competenc*"[Title/Abstract] OR "socio emotional competenc*"[Title/Abstract] OR "emotional abilit*"[Title/Abstract] OR "socioemotional abilit*"[Title/Abstract] OR "socio emotional abilit*"[Title/Abstract] OR "Emotional Intelligence"[Title/Abstract] OR "socio-emotional intelligence"[Title/Abstract] OR "emotional education"[Title/Abstract] OR "emotional learning"[Title/Abstract] OR "socioemotional learning"[Title/Abstract] OR "socio-emotional learning"[Title/Abstract] OR "emotional regulation"[Title/Abstract] OR "self-control"[Title/Abstract] OR "expressed emotion*"[Title/Abstract] OR "self-concept"[Title/Abstract] OR ("Emotional Regulation"[MeSH Major Topic:noexp] OR "Self-Control"[MeSH Major Topic:noexp] OR "Expressed Emotion"[MeSH Major Topic:noexp] OR "Emotional Intelligence"[MeSH Major Topic:noexp] OR "Self Concept"[MeSH Major Topic:noexp])) |
| #2 | ("undergraduat*"[Title/Abstract] OR "postgraduat*"[Title/Abstract] OR "graduat*"[Title/Abstract] OR "universit*"[Title/Abstract] OR "postsecondary"[Title/Abstract] OR "college"[Title/Abstract] OR "higher education"[Title/Abstract] OR "tertiary education"[Title/Abstract] OR "bachelor*"[Title/Abstract] OR "Professionalism"[Title/Abstract] OR ("Universities"[MeSH Major Topic:noexp] OR "education, medical"[MeSH Major Topic:noexp] OR "education, nursing"[MeSH Major Topic:noexp] OR "education, graduate"[MeSH Major Topic:noexp] OR "students, health occupations"[MeSH Major Topic:noexp] OR "Professionalism"[MeSH Major Topic:noexp]))) |
| #3 | ("addiction"[Title/Abstract] OR "substance"[Title/Abstract] OR "abuse"[Title/Abstract] OR "dependen*"[Title/Abstract] OR "alcohol"[Title/Abstract] OR "binge"[Title/Abstract] OR "smoke"[Title/Abstract] OR "gambl*"[Title/Abstract] OR "deaf*"[Title/Abstract] OR "posttraum*"[Title/Abstract] OR "trauma*"[Title/Abstract] OR "depressi*"[Title/Abstract] OR "disorder"[Title/Abstract] OR "bulimic"[Title/Abstract] OR "hyperactivity"[Title/Abstract] OR "suicid*"[Title/Abstract] OR "psychiatric*"[Title/Abstract] OR "patholog*"[Title/Abstract] OR "syndrome"[Title/Abstract] OR "autis*"[Title/Abstract] OR "schizophreni*"[Title/Abstract] OR "psychopath*"[Title/Abstract] OR "bipolar"[Title/Abstract] OR "self injur*"[Title/Abstract] OR "self-harm"[Title/Abstract] OR "Harassment"[Title/Abstract] OR "Stalking"[Title/Abstract] OR "Cyberbullying"[Title/Abstract] OR "diagnostic*"[Title/Abstract] OR "agressi*"[Title/Abstract] OR "violence"[Title/Abstract] OR "maltreatment"[Title/Abstract] OR "sexual*"[Title/Abstract] OR "sextortion"[Title/Abstract] OR "sexting"[Title/Abstract] OR "spiritual*"[Title/Abstract] OR "victim*"[Title/Abstract] OR "child*"[Title/Abstract] OR "parent*"[Title/Abstract] OR "couple"[Title/Abstract] OR "cultural"[Title/Abstract] OR "insomnia"[Title/Abstract] OR "stress"[Title/Abstract] OR "distress"[Title/Abstract] OR "anxiety"[Title/Abstract] OR "aggress*"[Title/Abstract])) |
| #4 | (#1 AND #2) NOT #3 |
| #5 | FILTER: (2000:2024[pdat]) |
| #6 | FILTER: (adult[Filter] OR middleaged[Filter]) |
| #7 | FILTER: (catalan[Filter] OR english[Filter] OR spanish[Filter]) |
| #8 | ALERTS ACTIVATED: Yes |

**d.** ERIC search strategy

| Step | Search terms |
| --- | --- |
| #1 | TI ( "emotional skill*" OR "socioemotional skill*" OR "socio-emotional skill*" OR "emotional competenc*" OR "socioemotional competenc*" OR "socio-emotional competenc*" OR "emotional abilit*" OR "socioemotional abilit*" OR "socio-emotional abilit*" OR "emotional intelligence" OR "socioemotional intelligence" OR "socio-emotional intelligence" OR "emotional education" OR "socioemotional education" OR "socio-emotional education" OR "emotional learning" OR "socioemotional learning" OR "socio-emotional learning" OR "self-concept" OR "self-control" ) OR AB ( "emotional skill*" OR "socioemotional skill*" OR "socio-emotional skill*" OR "emotional competenc*" OR "socioemotional competenc*" OR "socio-emotional competenc*" OR "emotional abilit*" OR "socioemotional abilit*" OR "socio-emotional abilit*" OR "emotional intelligence" OR "socioemotional intelligence" OR "socio-emotional intelligence" OR "emotional education" OR "socioemotional education" OR "socio-emotional education" OR "emotional learning" OR "socioemotional learning" OR "socio-emotional learning" OR "self-concept" OR "self-control" ) OR KW ( "emotional skill*" OR "socioemotional skill*" OR "socio-emotional skill*" OR "emotional competenc*" OR "socioemotional competenc*" OR "socio-emotional competenc*" OR "emotional abilit*" OR "socioemotional abilit*" OR "socio-emotional abilit*" OR "emotional intelligence" OR "socioemotional intelligence" OR "socio-emotional intelligence" OR "emotional education" OR "socioemotional education" OR "socio-emotional education" OR "emotional learning" OR "socioemotional learning" OR "socio-emotional learning" OR "self-concept" OR "self-control" ) OR (DE "Self Concept" OR DE "Self Control" DE "Emotional Intelligence") |
| #2 | TI ( Undergraduat* OR Postgraduat* OR graduat* OR universit* OR postsecondary OR college OR "higher education" OR "tertiary education" OR bachelor* OR professionalism ) OR AB ( Undergraduat* OR Postgraduat* OR graduat* OR universit* OR postsecondary OR college OR "higher education" OR "tertiary education" OR bachelor* OR professionalism ) OR KW ( Undergraduat* OR Postgraduat* OR graduat* OR universit* OR postsecondary OR college OR "higher education" OR "tertiary education" OR bachelor* OR professionalism ) OR (DE "Undergraduate Students" OR DE "Undergraduate Study" OR DE "Graduate Medical Education" OR DE "College Students" OR DE "Higher Education" OR DE "Bachelors Degrees") |
| #3 | TI ( addiction OR substance OR abuse OR dependen* OR alcohol OR binge OR smoke OR gambl* OR deaf* OR posttraum* OR trauma* OR depressi* OR disorder OR bulimic OR hyperactivity OR suicid* OR psychiatric* OR patholog* OR syndrome OR autis* OR schizophreni* OR psychopath* OR bipolar OR self-injur* OR self-harm OR Harassment OR Stalking OR Cyberbullying OR Diagnostic* OR agressi* OR violence OR maltreatment OR sex* OR spiritual* OR victim* OR child* OR parent* OR couple OR cultural OR insomnia OR stress OR distress OR anxiety OR aggress* ) NOT AB ( addiction OR substance OR abuse OR dependen* OR alcohol OR binge OR smoke OR gambl* OR deaf* OR posttraum* OR trauma* OR depressi* OR disorder OR bulimic OR hyperactivity OR suicid* OR psychiatric* OR patholog* OR syndrome OR autis* OR schizophreni* OR psychopath* OR bipolar OR self-injur* OR self-harm OR Harassment OR Stalking OR Cyberbullying OR Diagnostic* OR agressi* OR violence OR maltreatment OR sex* OR spiritual* OR victim* OR child* OR parent* OR couple OR cultural OR insomnia OR stress OR distress OR anxiety OR aggress* ) NOT KW ( addiction OR substance OR abuse OR dependen* OR alcohol OR binge OR smoke OR gambl* OR deaf* OR posttraum* OR trauma* OR depressi* OR disorder OR bulimic OR hyperactivity OR suicid* OR psychiatric* OR patholog* OR syndrome OR autis* OR schizophreni* OR psychopath* OR bipolar OR self-injur* OR self-harm OR Harassment OR Stalking OR Cyberbullying OR Diagnostic* OR agressi* OR violence OR maltreatment OR sex* OR spiritual* OR victim* OR child* OR parent* OR couple OR cultural OR insomnia OR stress OR distress OR anxiety OR aggress* ) |
| #4 | (#1 AND #2) NOT #3 |
| #5 | FILTER: Published Date: 20000101-20240126 |
| #6 | FILTER: Academic (Peer-Reviewed) Journals |
| #7 | FILTER: Education Level: Higher Education, Postsecondary Education, Two Year Colleges |
| #8 | FILTER: Language: English, Spanish; Castilian |
| #9 | ALERTS ACTIVATED: Yes |

**e.** Grey literature search strategy

| Search characteristics | | Research descriptors and expressions | |
| --- | --- | --- | --- |
|  |  | Exposure | Population |
| Sources | Google scholar and Google | emotional skill* | graduat* |
| Where the words occur | Anywhere in the publication | emotional competenc* | university* |
| Sorted by | Relevance | emotional ability* | postsecondary |
| Published between | January 2000 to January 2024 (included) | emotional intelligence | college |
| Reviewed the first | 150 results of each year | emotional education | higher education |
| Limits applied | No | emotional learning | tertiary education |
| Alerts activated | Yes |  | bachelor |
|  |  |  | professionalism |
