# Supplementary material for: Strategies to assess and promote the socio-emotional competencies of university students in the socio-educational and healthcare fields: A scoping review
Source: PLoS One. 2025 May 22;20(5):e0324531. doi: 10.1371/journal.pone.0324531 (PMC12097715; doi:10.1371/journal.pone.0324531)
Supplement: S3 Table — (DOCX) [file pone.0324531.s003.docx]

**S3 Table.** Data extraction protocol

| **Information of interest** | **Explanation** |
| --- | --- |
| **Information data** |  |
| Author | The individual or group of researchers responsible for conducting and publishing the study. |
| Publication year | The year in which the study was published. |
| **Data** | **Explanation** |
| **Information data** |  |
| Author | The individual or group of researchers responsible for conducting and publishing the study. |
| Publication year | The year in which the study was published. |
| Geographical origin of the sample | The country or region from which the participants were recruited. |
| Study field | The academic or professional discipline in which the study is conducted, specifically whether it belongs to the socio-educational, healthcare, or both fields. |
| Educational level | The academic stage of the participants, categorised as undergraduate, graduate, or postgraduate. |
| University degree | The specific degree programme in which the participants are enrolled. |
| **Methodological data** |  |
| Research design | The methodological approach used in the study. |
| Sampling technique | The method used to select participants for the study. |
| Purpose of the study | The main objective of the study, classified into one of the following categories: measurement studies (i.e., studies aimed at developing, adapting and/or testing SECs measures); descriptive and correlational studies (i.e., studies with the aim of describing or examining associations between SECs and other variables); and intervention studies (i.e., studies specifically tailored to evaluate intervention programmes for the promotion of SECs). |
| **Substantive data** |  |
| Theoretical SECs framework | The conceptual framework or model used to define and understand SECs. |
| Theoretical SECs reference | The full reference of the study. |
| SECs measures | The tools, instruments, or assessment methods used to evaluate participants' SECs. |
| SECs components assessed | The specific SECs measured in the study. |
| Form of administration | The method used to administer the assessment or data collection instrument. |
| Validity indicators | The evidence supporting the validity of the measurement tools used to assess SECs in the study |
| Reliability indicators | The statistical methods used to assess the consistency and reliability of SECs assessment instruments. |
| Promoted SECs | The SECs explicitly targeted and developed through the intervention or programme. |
| SEC are the primary or secondary objective of the intervention | Whether SECs were the main focus (primary objective) or an additional component (secondary objective) of the intervention. |
| Modality | Whether the intervention was integrated into formal academic curricula or conducted as an extracurricular activity. |
| Number of sessions, hours and frequency | The total number of sessions, the duration of each session, and how frequently they occurred within the intervention period. |
| Teaching strategies | The pedagogical approaches and techniques employed to develop SECs. |
| Prerequisites | The necessary conditions or requirements established before implementing the intervention. |
| Main results on SECs (i.e., efficacy and effectiveness) | The key findings related to the impact of the intervention on SECs, including its effectiveness and overall outcomes. |
