# Supplementary material for: Strategies to assess and promote the socio-emotional competencies of university students in the socio-educational and healthcare fields: A scoping review
Source: PLoS One. 2025 May 22;20(5):e0324531. doi: 10.1371/journal.pone.0324531 (PMC12097715; doi:10.1371/journal.pone.0324531)
Supplement: S1 Text — (DOCX) [file pone.0324531.s005.docx]

**S1 Text.** References of included studies

**a.** References of measure studies included in the review

1. Arifin WN, Yusoff MSB. Confirmatory Factor Analysis of the University Sains Malaysia Emotional Quotient Inventory Among Medical Students in Malaysia. SAGE Open. 2016;6: e1–e22. doi:10.1177/2158244016650240
2. Balzarotti S. The emotion regulation questionnaire: Factor structure and measurement invariance in an Italian sample of community dwelling adults. Curr Psychol A J Divers Perspect Divers Psychol Issues. 2021;40: 4918–4929. 10.1007/s12144-019-00426-3
3. Brannick MT, Wahi MM, Arce M, Johnson HAH-A, Nazian S, Goldin SB. Comparison of trait and ability measures of emotional intelligence in medical students. Med Educ. 2009;43: 1062–1068. doi:10.1111/j.1365-2923.2009.03430.x
4. Cabral J, Brandão T, Lamela D, Matos PM. The psychometric properties of a Portuguese version of the Trait-Meta Mood Scale: An attachment framework. J Psychopathol Behav Assess. 2021;43: 162–173. https: 10.1007/s10862-020-09817-4
5. Callwood A, Cooke D, Allan H. Developing and piloting the multiple mini-interview in pre-registration student midwife selection in a UK setting. Nurse Educ Today. 2014;34: 1450–1454. doi:10.1016/j.nedt.2014.04.023
6. Carvalho VS, Guerrero E, Chambel MJ, González-Rico P. Psychometric properties of WLEIS as a measure of emotional intelligence in the Portuguese and Spanish medical students. Eval Program Plann. 2016;58: 152–159. doi:10.1016/j.evalprogplan.2016.06.006
7. Chew HSJ, Ang WHD, Rusli KDB, Liaw SY, Ang NKE, Lau Y. Development and psychometric properties testing of the Non-Cognitive Skills Scale for nursing students. Nurse Educ Today. 2024;132: 105996. doi:10.1016/j.nedt.2023.105996
8. Cowin L. Measuring nurses’ self-concept. West J Nurs Res. 2001;23: 313–325. doi:10.1177/01939450122045177
9. Cronin L, Allen J, Ellison P, Marchant D, Levy A, Harwood C. Development and Initial Validation of the Life Skills Ability Scale for Higher Education Students. Stud High Educ. 2019;46: 1011–1024. doi:10.1080/03075079.2019.1672641
10. de Souza RR, Faiad C, Rueda FJM. Construction and validity evidence of a Socioemotional Skills Scale for University Students. Avaliação Psicológica. 2021;20: 445–454. Available: https://www.proquest.com/scholarly-journals/construction-validity-evidence-socioemotional/docview/2807990091/se-2?accountid=14777
11. Dimitrijević AA, Marjanović ZJ, Starčević J. The vocabulary of emotions test (Vet): Psychometric properties of the serbian version. Psihol Teme. 2020;29: 135–150. doi:10.31820/pt.29.1.8
12. Dominguez-Lara S, Campos-Uscanga Y. Internal structure of a brief emotional intelligence scale in Mexican health sciences students. Educ Medica. 2021;22: 262–266. doi:10.1016/j.edumed.2019.10.010
13. Edo-Gual M, Tomás-Sábado J, Gómez-Benito J, Monforte-Royo C, Aradilla-Herrero A. Spanish Adaptation of the Frommelt Attitude Toward Care of the Dying Scale (FATCOD-S) in Nursing Undergraduates. Omega. 2018;78: 120–142. doi:10.1177/0030222816688294
14. Faria L, Santos NL. Validation of the Emotional Skills and Competence Questionnaire (ESCQ) in the Portuguese academic context. Psihol Obz / Horizons Psychol. 2009;18: 55–71.
15. González R, Custodio JB, Abal FJP. Psychometric properties of the Trait Meta-Mood Scale-24 in argentinian university students. Psicogente. 2020;23: 1–26. doi:10.17081/psico.23.44.3469
16. Haavisto E, Hupli M, Hahtela N, Heikkilä A, Huovila P, Moisio EL, et al. Structure and Content of a New Entrance Exam to Select Undergraduate Nursing Students. Int J Nurs Educ Scholarsh. 2019;16: 20180008. doi:10.1515/ijnes-2018-0008
17. Naeem N, Muijtjens A. Validity and reliability of bilingual English-Arabic version of Schutte self report emotional intelligence scale in an undergraduate Arab medical student sample. Med Teach. 2015;37: S20–S26. doi:10.3109/0142159X.2015.1006605
18. Pienimaa A, Talman K, Vierula J, Laakkonen E, Haavisto E. Development and psychometric evaluation of the Emotional Intelligence Test (EMI-T) for social care and healthcare student selection. J Adv Nurs. 2023;79: 850–863. https://doi.org/10.1111/jan.15557
19. Reivan-Ortiz GG, Rodas PEO, Ortiz PNR. A brief version of the Difficulties in Emotion Regulation Scale (DERS): Validity evidence in Ecuadorian population. Int J Psychol Res. 2020;13: 14–24. https://doi.org/10.21500/20112084.4325
20. Rodríguez-Sabiote C, Ibáñez-Cubillas P, López-Rodríguez S, Álvarez-Rodríguez J. Psychometric properties of the Spanish version of the Cognitive Emotional Regulation Difficulties Questionnaire (CERQ) in higher education students in times of COVID-19. Front Psychol. 2021;12: Article 695147. https://doi.org/10.3389/fpsyg.2021.695147
21. Roll M, Canham L, Salamh P, Covington K, Simon C, Cook C. A novel tool for evaluating non-cognitive traits of doctor of physical therapy learners in the United States. J Educ Eval Health Prof. 2018;15: Article 19. doi:10.3352/jeehp.2018.15.19
22. Snowden A, Watson R, Stenhouse R, Hale C. Emotional Intelligence and Nurse Recruitment: Rasch and confirmatory factor analysis of the trait emotional intelligence questionnaire short form. J Adv Nurs. 2015;71: 2936–2949. doi:10.1111/jan.12746
23. Zysberg L, Levy A, Zisberg A. Emotional Intelligence in Applicant Selection for Care-Related Academic Programs. J Psychoeduc Assess. 2011;29: 27–38. https://doi.org/10.1177/0734282910365059

**b.** References of intervention studies included in the review

1. Abdulqadir MA, Samein LH, Jasim MA, Ali MH, Abbas AH. An Evaluation of the Impact of Teaching Emotional Intelligence Components on the Emotional Intelligence of Medical and Nursing StudentsAn Evaluation of the Impact of Teaching Emotional Intelligence Components on the Emotional Intelligence of Medical and N. Int J Body, Mind Cult. 2022;9: 207–217. doi:10.22122/ijbmc.v9i3.406
2. Abe K, Evans P, Austin EJ, Suzuki Y, Fujisaki K, Niwa M, et al. Expressing one’s feelings and listening to others increases emotional intelligence: A pilot study of Asian medical students. BMC Med Educ. 2013;13: Article 82. doi:10.1186/1472-6920-13-82
3. Antoun J, Bou Akl I, Halabi Z, Bou Khalil P, Romani M. Effect of Balint seminars training on emotional intelligence and burnout among internal medicine residents. Health Educ J. 2020;79: 802–811. doi:10.1177/0017896920911684
4. Arch JJ, Craske MG. Mechanisms of mindfulness: Emotion regulation following a focused breathing induction. Behav Res Ther. 2006;44: 1849–1858. doi:10.1016/j.brat.2005.12.007
5. Basari S, Güneyli A, Yiltas GL, Kahveci G. The impact of bibliotherapy training on social-emotional skills, reading interest and reading motivation. Cyprus Turkish J Psychiatry Psychol. 2023;5: 13–24. doi:10.35365/ctjpp.23.1.02
6. Beauvais AM, Özbaş AA, Wheeler K. End-of-life psychodrama: Influencing nursing students’ communication skills, attitudes, emotional intelligence and self-reflection. J Psychiatr Nurs. 2019;10: 103–110. doi:10.14744/phd.2019.96636
7. Bonazza NA, Cabell GH, Cheah JW, Taylor DC. Effect of a novel healthcare leadership program on leadership and emotional intelligence. Healthc Manag Forum. 2021;34: 272–277. doi:10.1177/08404704211036667
8. Bonvicini KA, Perlin MJ, Bylund CL, Carroll G, Rouse RA, Goldstein MG. Impact of communication training on physician expression of empathy in patient encounters. Patient Educ Couns. 2009;75: 3–10. doi:10.1016/j.pec.2008.09.007
9. Bowling AM, Underwood PW. Effect of simulation on knowledge, self-confidence, and skill performance in the USA: A quasi-experimental study. Nurs Health Sci. 2016;18: 292–298. doi:10.1111/nhs.12267
10. Brison C, Zech E, Jaeken M, Priels J-M, Verhofstadt L, Van Broeck N, et al. Encounter groups: Do they foster psychology students’ psychological development and therapeutic attitudes? Pers Exp Psychother. 2015;14: 83–99. https://dx.doi.org/10.1080/14779757.2014.991937
11. Buckley K, Bowman B, Raney E, Afolabi T, Fettkether RM, Larson S, et al. Enhancing the emotional intelligence of student leaders within an accelerated pharmacy program. Am J Pharm Educ. 2020;84: 8056. doi:10.5688/ajpe8056
12. Caballero-García PA, Sánchez Ruiz S. Creativity and life satisfaction in Spanish university students. Effects of an emotionally positive and creative program. Front Psychol. 2021;12: 746154. https://doi.org/10.3389/fpsyg.2021.746154
13. Choi Y, Song E, Oh E. Effects of teaching communication skills using a video clip on a smart phone on communication competence and emotional intelligence in nursing students. Arch Psychiatr Nurs. 2015;29: 90–95. doi:10.1016/j.apnu.2014.11.003
14. Choi Y-JJ. Evaluation of a program on self-esteem and ego-identity for Korean nursing students. Nurs Health Sci. 2016;18: 387–392. doi:10.1111/nhs.12281
15. Christiansen B, Jensen K. Emotional learning within the framework of nursing education. Nurse Educ Pract. 2008;8: 328–334. doi:10.1016/j.nepr.2008.01.003
16. Cole JD, Ballou JM, Declue A, Ruble MJ, Noble M, Euler M, et al. The impact of leadership program formatting on perceived development within pharmacy cohorts. Am J Pharm Educ. 2023;87: 364–371. doi:10.5688/ajpe9005
17. Cunico L, Sartori R, Marognolli O, Meneghini AM. Developing empathy in nursing students: A cohort longitudinal study. J Clin Nurs. 2012;21: 2016–2025. doi:10.1111/j.1365-2702.2012.04105.x
18. DasGupta S, Charon R. Personal Illness Narratives: Using Reflective Writing to Teach Empathy. Acad Med J Assoc Am Med Coll. 2004;79: 351–356. https://doi.org/10.1097/00001888-200404000-00013
19. Donisi V, Perlini C, Mazzi MA, Rimondini M, Garbin D, Ardenghi S, et al. Training in communication and emotion handling skills for students attending medical school: Relationship with empathy, emotional intelligence, and attachment style. Patient Educ Couns. 2022;105: 2871–2879. doi:10.1016/j.pec.2022.05.015
20. Duran A, Donelan C, Bowman Peterson J, Gladding SP, Weissmann P, Roth CS. Communicating value to patients-a high-value care communication skills curriculum. Postgrad Med. 2021;133: 231–236. doi:10.1080/00325481.2020.1807728
21. Farver CF, Smalling S, Stoller JK, Bennett JW. Developing leadership competencies among medical trainees: Five-year experience at the Cleveland Clinic with a chief residents’ training course. Australas Psychiatry. 2016;24: 499–505. doi:10.1177/1039856216632396
22. Fincias PT, González SS. Benefits of intergenerational programs in the development of emotional competencies. Estud Pedagog. 2021;47: 273–290. doi:10.4067/S0718-07052021000300273
23. Fletcher I, Leadbetter P, Curran A, O’Sullivan H. A pilot study assessing emotional intelligence training and communication skills with 3rd year medical students. Patient Educ Couns. 2009;76: 376–379. doi:10.1016/j.pec.2009.07.019
24. Galal S, Carr-Lopez S, Seal CR, Scott AN, Lopez C. Development and assessment of social and emotional competence through simulated patient consultations. Am J Pharm Educ. 2012;76: Article 132. doi:10.5688/ajpe767132
25. Ghetti C, Chang J, Gosman G. Burnout, Psychological Skills, and Empathy: Balint Training in Obstetrics and Gynecology Residents. J Grad Med Educ. 2009;1: 231–235. doi:10.4300/jgme-d-09-00049.1
26. Gorgas DL, Greenberger S, Bahner DP, Way DP. Teaching emotional intelligence: A control group study of a brief educational intervention for emergency medicine residents. West J Emerg Med. 2015;16: 899–906. doi:10.5811/westjem.2015.8.27304
27. Goroshit M, Hen M. Emotional Intelligence: A Stable Change? Int J Teach Learn High Educ. 2012;24: 31–42.
28. Goudarzian AH, Nesami MB, Sedghi P, Gholami M, Faraji M, Hatkehlouei MB. The Effect of Self-Care Education on Emotional Intelligence of Iranian Nursing Students: A Quasi-experimental Study. J Relig Health. 2019;58: 589–598. doi:10.1007/s10943-017-0537-3
29. Gunasingha RMKD, Lee H-J, Zhao C, Clay A. Conflict resolution styles and skills and variation among medical students. BMC Med Educ. 2023;23: Article 246. doi:10.1186/s12909-023-04228-x
30. Hen M, Goroshit M. Emotional competencies in the education of mental health professionals. Soc Work Educ. 2011;30: 811–829. doi: 10.1080/02615479.2010.515680
31. Hurley J, Hutchinson M, Kozlowski D, Gadd M, van Vorst S. Emotional intelligence as a mechanism to build resilience and non-technical skills in undergraduate nurses undertaking clinical placement. Int J Ment Health Nurs. 2020;29: 47–55. doi:10.1111/inm.12607
32. Imperato A, Strano-Paul L. Impact of reflection on empathy and emotional intelligence in third-year medical students. Acad Psychiatry. 2021;45: 350–353. https://doi.org/10.1007/s40596-020-01371-1
33. Jiménez-Rodríguez D, Molero Jurado MM, Pérez-Fuentes MC, Arrogante O, Oropesa-Ruiz NF, Gázquez-Linares JJ. The Effects of a Non-Technical Skills Training Program on Emotional Intelligence and Resilience in Undergraduate Nursing Students. Healthc. 2022;10: Article 866. doi:10.3390/healthcare10050866
34. Johnsen J-AK, Borit M, Stangvaltaite-Mouhat L. Using storytelling in undergraduate dental education: Students’ experiences of emotional competence training. Eur J Dent Educ. 2023;27: 793–801. doi:10.1111/eje.12868
35. Kelly T, Thompson JD, Surjan Y, Rinks M, Warren-Forward H. Lived experiences of first-year radiation therapy students communicating with patients and radiation therapists: A qualitative review using interpretative phenomenological analysis. Radiography. 2022;28: 168–173. doi:10.1016/j.radi.2021.10.002
36. Kirkpatrick AJ, Cantrell MA, Smeltzer SC. Palliative care knowledge and self-awareness in active and observing undergraduate nursing students after end-of-life simulation. Int J Palliat Nurs. 2020;26: 133–142. doi:10.12968/ijpn.2020.26.3.133
37. Kneese G, Barrera S, Castillo J, Garcia M, Ryden A, White A, et al. ScribeMD: Mixed-methods analysis of a pen pal program in undergraduate medical education. Med Teach. 2020;42: 316–324. doi:10.1080/0142159X.2019.1686134
38. Krasner MS, Epstein RM, Beckman H, Suchman AL, Chapman B, Mooney CJ, et al. Association of an educational program in mindful communication with burnout, empathy, and attitudes among primary care physicians. JAMA - J Am Med Assoc. 2009;302: 1284–1293. doi:10.1001/jama.2009.1384
39. Latif DA. Using emotional intelligence in the planning and implementation of a management skills course. Pharm Educ. 2004;4: 81–89. doi:10.1080/15602210410001701685
40. LeBlanc S, Uzun B, Pourseied K, Mohiyeddini C. Effect of an emotion regulation training program on mental well-being. Int J Group Psychother. 2017;67: 108–123.
41. Lefroy J, Brosnan C, Creavin S. Some like it hot: Medical student views on choosing the emotional level of a simulation. Med Educ. 2011;45: 354–361. doi:10.1111/j.1365-2923.2010.03881.x
42. Lim JY, Kim MA, Kim SY, Kim EJ, Lee JE, Ko YK. The effects of a cognitive-behavioral therapy on career attitude maturity, decision making style, and self-esteem of nursing students in Korea. Nurse Educ Today. 2010;30: 731–736. doi:10.1016/j.nedt.2010.01.014
43. Lust E, Moore FC. Emotional intelligence instruction in a pharmacy communications course. Am J Pharm Educ. 2006;70: Article 6. doi:10.5688/aj700106
44. McConville SA, Lane AM. Using on-line video clips to enhance self-efficacy toward dealing with difficult situations among nursing students. Nurse Educ Today. 2006;26: 200–208. doi:10.1016/j.nedt.2005.09.024
45. Mortari L. Emotion and Education: Reflecting on the Emotional Experience Emotion and Education. Eur J Educ Res. 2015;4: 157–176. doi:doi: 10.12973/eu-jer.4.4.157
46. Orak RJ, Farahani MA, Kelishami FG, Seyedfatemi N, Banihashemi S, Havaei F. Investigating the effect of emotional intelligence education on baccalaureate nursing students’ emotional intelligence scores. Nurse Educ Pract. 2016;20: 64–69. doi:10.1016/j.nepr.2016.05.007
47. Pades Jiménez A, García-Buades ME, Riquelme I. Development of emotional intelligence and assertiveness in physiotherapy students and effects of clinical placements. Physiother Theory Pract. 2023;39: 72–79. doi:10.1080/09593985.2021.2005200
48. Parks MH, Chen CK, Haygood CD, Mcgee ML. Altered emotional intelligence through a health disparity curriculum: Early results. J Health Care Poor Underserved. 2019;30: 1486–1498. doi:10.1353/hpu.2019.0091
49. Price ET, Coverley CR, Arrington AK, Nfonsam VN, Morris-Wiseman L, Riall TS. Are We Making an Impact? A Qualitative Program Assessment of the Resident Leadership, Well-being, and Resiliency Program for General Surgery Residents. J Surg Educ. 2020;77: 508–519. doi:10.1016/j.jsurg.2019.12.003
50. Puffer KA, Pence KG, Ferry AE. A feasibility study on an ultra-brief intervention for improving freshmen’s emotional intelligence. J Intell. 2021;9: Article 36. https://doi.org/10.3390/jintelligence9030036
51. Raatikainen E, Rauhala LA, Mäenpää S. An Educational Intervention Focused on Teaching Qualified Empathy to Social Work Students in Finland. J Appl Res High Educ. 2022;14: 409–423. doi:10.1108/JARHE-11-2020-0404
52. Redondo-Rodríguez C, Becerra-Mejías JA, Gil-Fernández G, Rodríguez-Velasco FJ. Influence of Gamification and Cooperative Work in Peer, Mixed and Interdisciplinary Teams on Emotional Intelligence, Learning Strategies and Life Goals That Motivate University Students to Study. Int J Environ Res Public Health. 2023;20: Article 547. doi:10.3390/ijerph20010547
53. Reshetnikov VA, Tvorogova ND, Hersonskiy II, Sokolov NA, Petrunin AD, Drobyshev DA. Leadership and Emotional Intelligence: Current Trends in Public Health Professionals Training. Front Public Heal. 2020;7: Article 413. doi:10.3389/fpubh.2019.00413
54. Ribeiro RM, Bernardini Bragiola J V., Eid LP, Helú RC, da Cruz SCA, Pompeo DA. Impact of an intervention through Facebook to strengthen Self-esteem in nursing students. Rev Lat Am Enfermagem. 2020;28: e3237. doi:10.1590/1518-8345.3215.3237
55. Rosa G, Riberas G, Navarro-Segura L, Vilar J. El coaching como herramienta de trabajo de la competencia emocional en la formación de estudiantes de educación social y trabajo social de la Universidad Ramón Llull, España. Form Univ. 2015;8: 77–90. doi:10.4067/S0718-50062015000500009
56. Rothe EM, Bonnin R. Utilizing psychodynamic principles to teach professionalism to medical students through an innovative curriculum. Psychodyn Psychiatry. 2020;48: 477–497. https://doi.org/10.1521/pdps.2020.48.4.477
57. Rowland Z, Wenzel M, Kubiak T. Effects of an ultra-brief computer-based mindfulness training on mindfulness and self-control: A randomised controlled trial using a 40-day ecological momentary assessment. Mindfulness (N Y). 2019;10: 2312–2326. doi:10.1007/s12671-019-01204-9
58. Seow HY, Wu MHL, Mohan M, Mamat NH, Kutzsche HE, Pau A. The effect of transactional analysis training on emotional intelligence in health professions students. BMC Med Educ. 2022;22: Article 383. doi:10.1186/s12909-022-03455-y
59. Shrivastava S, Martinez J, Coletti DJ, Fornari A. Interprofessional Leadership Development: Role of Emotional Intelligence and Communication Skills Training. MedEdPORTAL  J Teach Learn Resour. 2022;18: 11247. doi:10.15766/mep_2374-8265.11247
60. Song EY, Chuang J, Frakes JM, Dilling T, Quinn JF, Rosenberg S, et al. Developing a dedicated leadership curriculum for radiation oncology residents. J Cancer Educ. 2022;37: 1446–1453. https://doi.org/10.1007/s13187-021-01980-w
61. Sousa, C. R., & da Costa Padovani, R. (2021). Assertive skills: a comparison of two group interventions with Brazilian university students. *Psicologia: Reflexao e Critica*, *34*(27), 1–9. https://doi.org/10.1186/s41155-021-00188-7
62. Watford TS, Stafford J. The impact of mindfulness on emotion dysregulation and psychophysiological reactivity under emotional provocation. Psychol Conscious Theory, Res Pract. 2015;2: 90–109. doi:10.1037/cns0000039
63. Yiu JW, Mak WWS, Ho WS, Chui YY. Effectiveness of a knowledge-contact program in improving nursing students’ attitudes and emotional competence in serving people living with HIV/AIDS. Soc Sci Med. 2010;71: 38–44. https://doi.org/10.1016/j.socscimed.2010.02.045
64. Yoong SQ, Schmidt LT, Devi KM, Zhang H. Using palliative and end-of-life simulation to enhance pre-licensure nursing students’ emotional intelligence, palliative care knowledge and reflective abilities: A single group, pretest-posttest study. Nurse Educ Today. 2023;130: Article 105923. doi:10.1016/j.nedt.2023.105923

**c.** References of correlational studies included in the review

1. Abdollahpour I, Nedjat S, Besharat MA, Hosseini B, Salimi Y. Emotional intelligence: A comparison between medical and non-medical students. Iran J Public Health. 2016;45: 214–222.
2. Abella MC, Carreira BM, Rodríguez DA, Carballeira Abella M, Marrero Carreira B, Abrante Rodriguez D. Emotional intelligence and psychological adjustment in students: Academic level and field of study. Univ Psychol. 2019;18: 1–14. doi:10.11144/Javeriana.upsy18-4.ieap
3. Abu Alkhayr L, Alshaikh R, Alghamdi L, Alshaikh A, Somaa F, Bokhari FA. Is emotional intelligence linked with academic achievement? The first TEIQue-SF study in a sample of Saudi medical rehabilitation students. Ann Med Surg. 2022;78: Article 103726. doi:10.1016/j.amsu.2022.103726
4. Adamik M. Does mindfulness moderate the relationship between self-reported emotional intelligence and facial expression recognition? Ann Psychol. 2018;21: 299–307. doi:10.18290/rpsych.2018.21.4-1
5. Aguilar-Luzón M c, Augusto Landa JM. Relationship between perceived emotional intelligence, personality and empathic behavior in nursing students. Behav Psychol. 2009;17: 351–364. Available: https://www.proquest.com/scholarly-journals/relación-entre-inteligencia-emocional-percibida/docview/622035973/se-2?accountid=14777
6. Ahmad I. Psychological Predictors of College Students Performance. Pakistan J Psychol Res. 2011;26: 87–103. https://doi.org/10.33824/PJPR.2020.35.1.6
7. Aithal AP, Kumar N, Gunasegeran P, Sundaram SM, Rong LZ, Prabhu SP. A survey-based study of emotional intelligence as it relates to gender and academic performance of medical students. Educ Heal. 2016;29: 255–258. doi:10.4103/1357-6283.204227
8. Akbarilakeh M, Naderi A, Arbabisarjou A. Critical thinking and emotional intelligence skills and relationship with students’ academic achievement. Prensa Med Argent. 2018;104: Article 1000280. doi:10.4172/0032-745X.1000280
9. Al Qamash MN, Altal SM, Jawaldeh FE. Dimensional common emotional intelligence for the student of higher education in princess alia college at the university of al balq’a applied university in jordan from the point of view of the students. Eur J Soc Sci. 2011;26: 87–97. Available: https://www.scopus.com/inward/record.uri?eid=2-s2.0-82055201371&partnerID=40&md5=d9ed3fceee8cf87ae1167e1ebc1eff21
10. Alipour N, Sangi S, Babamiri M, Arman P. Investigating the relationship between emotional intelligence and self-esteem with educational performance in paramedical students. Med Clin Pract. 2024;7: Article 100398. doi:10.1016/j.mcpsp.2023.100398
11. Alkhadher O. Emotional intelligence and psychological health in a sample of Kuwaiti college students. Percept Mot Skills. 2007;104: 923–936. https://dx.doi.org/10.2466/PMS.104.3.923-936
12. Almansour AM. The level of emotional intelligence among Saudi nursing students: A cross-sectional study. Belitung Nurs J. 2023;9: 471–477. doi:10.33546/bnj.2794
13. Almegewly WH, Rawdhan A, Saleh M, Alrimal M, Alasmari R, Alhamad S, et al. Correlation between emotional intelligence and academic achievement among undergraduate nursing students. Int J Africa Nurs Sci. 2022;17: Article 100491. doi:10.1016/j.ijans.2022.100491
14. Álvarez-Huerta P, Muela A, Larrea I. Cognitive reappraisal and creative self-concept among higher education students: A latent class analysis. Psychol Aesthetics, Creat Arts. 2023; Advanced online publication. https://doi.org/10.1037/aca0000567
15. Alvi T, Nadakuditi RL, Alotaibi TH, Aisha A, Ahmad MS, Ahmad S. Emotional intelligence and academic performance among medical students – a correlational study. Eur Rev Med Pharmacol Sci. 2023;27: 1230–1237. doi:10.26355/eurrev_202302_31355
16. Aneas Álvarez A, Carmona Rodríguez C, Lorenzo Ramírez N, Ferré Tobaruela M. Relationship between Prejudice, Emotional Competence and Coping Strategies in Social Education students. An exploratory study in three Spanish universities. Rev Lusofona Educ. 2023;60: 121–138. doi:10.24140/issn.1645-7250.rle60.08
17. Astra RL, Singg S. The role of self-esteem in affiliation. J Psychol. 2000;134: 15–22. doi:10.1080/00223980009600845
18. Augusto Landa JM, Aguilar-Luzón MC, Salguero MF. The role of perceived emotional intelligence and dispositional optimism/pessimism in social problem solving: A study of social work students. Electron J Res Educ Psychol. 2008;6: 363–382. Available: https://www.proquest.com/scholarly-journals/role-perceived-emotional-intelligence/docview/621967785/se-2?accountid=14777
19. Augusto Landa JM, López-Zafra E, Aguilar-Luzón MCM del C, de Ugarte MFS. Predictive validity of Perceived Emotional Intelligence on nursing students’ self-concept. Nurse Educ Today. 2009;29: 801–808. https://doi.org/10.1016/j.nedt.2009.04.004
20. Ayala G, Keren G. Relationship between emotional intelligence and burnout in nursing students. Acta Sci Paediatr. 2020;3: 10–18.
21. Babić Čikeš A, Tomašić Humer J. Ability and Trait Emotional Intelligence: Do They Contribute to the Explanation of Prosocial Behaviour? Eur J Investig Heal Psychol Educ. 2023;13: 964–974. doi:10.3390/ejihpe13060073
22. Bano Z, Pervaiz S. The Relationship Between Resilience, Emotional Intelligence and Their Influence on Psychological Wellbeing: A Study with Medical Students. Pakistan Armed Forces Med J. 2020;70: 390–394. Available: https://www.scopus.com/inward/record.uri?eid=2-s2.0-85105161556&partnerID=40&md5=e41f46f38ca03835607dd5ad2f9f6272
23. Barchard KA. Does emotional intelligence assist in the prediction of academic success? Educ Psychol Meas. 2003;63: 840–858. https://dx.doi.org/10.1177/0013164403251333
24. Batmaz M, Kendirkiran G, Kavurucu Ö. The effects of the education received by nursing students on their self-esteem and emotional intelligence: A 4-year longitudinal study. Perspect Psychiatr Care. 2022;58: 2088–2098. doi:10.1111/ppc.13035
25. Beauvais AM, Brady N, O’Shea ER, Griffin MTQ. Emotional intelligence and nursing performance among nursing students. Nurse Educ Today. 2011;31: 396–401. doi:10.1016/j.nedt.2010.07.013
26. Beierle SP, Kirkpatrick BA, Heidel RE, Russ A, Ramshaw B, McCallum RS, et al. Evaluating and Exploring Variations in Surgical Resident Emotional Intelligence and Burnout. J Surg Educ. 2019;76: 628–636. doi:10.1016/j.jsurg.2018.11.004
27. Benington MR, Hussey LC, Long JM. Emotional intelligence and successful completion of nursing courses in associate degree nursing students. Teach Learn Nurs. 2020;15: 186–189. doi:10.1016/j.teln.2020.03.003
28. Benson G, Martin L, Ploeg J, Wessel J. Longitudinal study of emotional intelligence, leadership, and caring in undergraduate nursing students. J Nurs Educ. 2012;51: 95–101. doi:10.3928/01484834-20120113-01
29. Bertram K, Randazzo J, Alabi N, Levenson J, Doucette JT, Barbosa P. Strong correlations between empathy, emotional intelligence, and personality traits among podiatric medical students: A cross-sectional study. Educ Heal. 2016;29: 186–194. doi:10.4103/1357-6283.204224
30. Blanchard C, Kravets V, Schenker M, Moore T. Emotional intelligence, burnout, and professional fulfillment in clinical year medical students. Med Teach. 2021;43: 1063–1069. doi:10.1080/0142159X.2021.1915468
31. Bourgeon L, Bensalah M, Vacher A, Ardouin J-CJ-C, Debien B. Role of emotional competence in residents’ simulated emergency care performance: a mixed-methods study. BMJ Qual. 2016;25: 364–371. doi:10.1136/bmjqs-2015-004032
32. Brannick MT, Grichanik M, Nazian SJ, Wahi M, Goldin SB. Emotional Intelligence and Medical School Performance: A Prospective Multivariate Study. Med Sci Educ. 2013;23: 628–636. doi:10.1007/BF03341690
33. Budler LC, Gosak L, Vrbnjak D, Pajnkihar M, Štiglic G. Emotional Intelligence among Nursing Students: Findings from a Longitudinal Study. Healthc. 2022;10: Article 2032. doi:10.3390/healthcare10102032
34. Carmichael M, Bridge P, Harriman A. Emotional intelligence development in radiation therapy students: a longitudinal study. J Radiother Pract. 2016;15: 45–53. doi:10.1017/S1460396915000461
35. Carr SE, Celenza A, Mercer AM, Lake F, Puddey IB. Predicting performance of junior doctors: Association of workplace based assessment with demographic characteristics, emotional intelligence, selection scores, and undergraduate academic performance. Med Teach. 2018;40: 1175–1182. doi:10.1080/0142159X.2018.1426840
36. Carr SE. Emotional intelligence in medical students: Does it correlate with selection measures? Med Educ. 2009;43: 1069–1077. doi:10.1111/j.1365-2923.2009.03496.x
37. Carvalho VS, Guerrero E, Chambel MJ. Emotional intelligence and health students’ well-being: A two-wave study with students of medicine, physiotherapy and nursing. Nurse Educ Today. 2018;63: 35–42. doi:10.1016/j.nedt.2018.01.010
38. Chan JCY, Hamamura T. Emotional Intelligence, Pain Knowledge, and Attitudes of Nursing Students in Hong Kong. Pain Manag Nurs. 2016;17: 159–168. doi:10.1016/j.pmn.2016.02.001
39. Chan JCY, Sit ENM, Lau WM. Conflict management styles, emotional intelligence and implicit theories of personality of nursing students: A cross-sectional study. Nurse Educ Today. 2014;34: 934–939. doi:10.1016/j.nedt.2013.10.012
40. Chatterjee MB, Baumann N, Koole SL. Feeling better when someone is alike: Poor emotion regulators profit from pro‐social values and priming for similarities with close others. J Pers. 2017;85: 841–851. doi:10.1111/jopy.12292
41. Chen H, Zhang MH. The relationship between basic psychological needs satisfaction and university students’ academic engagement: The mediating effect of emotional intelligence. Front Psychol. 2022;13: Article 917578. doi:10.3389/fpsyg.2022.917578
42. Chew B-H, Zain AM, Hassan F. The relationship between the social management of emotional intelligence and academic performance among medical students. Psychol Heal Med. 2015;20: 198–204. doi:10.1080/13548506.2014.913797
43. Christodoulakis A, Kritsotakis G, Gkorezis P, Sourtzi P, Tsiligianni I. Linking Learning Environment and Critical Thinking through Emotional Intelligence: A Cross-Sectional Study of Health Sciences Students. Healthc. 2023;11: Article 826. doi:10.3390/healthcare11060826
44. Christodoulakis A, Kritsotakis G, Linardakis M, Sourtzi P, Tsiligianni I. Emotional intelligence is more important than the learning environment in improving critical thinking. Med Teach. 2023;45: 708–716. doi:10.1080/0142159X.2023.2193305
45. Codier E, Odell E. Measured emotional intelligence ability and grade point average in nursing students. Nurse Educ Today. 2014;34: 608–612. doi:10.1016/j.nedt.2013.06.007
46. Codier EE, Kofoed NA, Peters JM. Graduate-entry non-nursing students: Is emotional intelligence the difference? Nurs Educ Perspect. 2015;36: 46–47. doi:10.5480/12-874.1
47. Cofer KD, Hollis RH, Goss L, Morris MS, Porterfield JR, Chu DI. Burnout is Associated With Emotional Intelligence but not Traditional Job Performance Measurements in Surgical Residents. J Surg Educ. 2018;75: 1171–1179. doi:10.1016/j.jsurg.2018.01.021
48. Coleman A, Oliveros AD. Reconceptualization of emotion regulation: strategy use, flexibility, and emotionality. Anxiety Stress Coping. 2020;33: 19–30. doi:10.1080/10615806.2019.1655641
49. Collins S. Emotional intelligence as a noncognitive factor in student registered nurse anesthetists. AANA J. 2013;81: 465–472.
50. Collins SB, Covrig D, Newman I. Q-factor emotional intelligence profiles as an area for development in graduate nurse anesthesia students. J Nurs Educ. 2014;53: 501–510. doi:10.3928/01484834-20140821-13
51. Cowin LS, Hengstberger-Sims C. New graduate nurse self-concept and retention: a longitudinal survey. Int J Nurs Stud. 2006;43: 59–70. doi:10.1016/j.ijnurstu.2005.03.004
52. Cuartero N, Tur AM. Emotional intelligence, resilience and personality traits neuroticism and extraversion: predictive capacity in perceived academic efficacy. Nurse Educ Today. 2021;102: Article 104933. doi:10.1016/j.nedt.2021.104933
53. Culha Y, Acaroglu R. The relationship amongst student nurses’ values, emotional intelligence and individualised care perceptions. Nurs Ethics. 2019;26: 2373–2383. doi:10.1177/0969733018796682
54. Czabanowska K, Malho A, Schröder-Bäck P, Popa D, Burazeri G. Do we develop public health leaders?- Association between public health competencies and emotional intelligence: A cross-sectional study. BMC Med Educ. 2014;14: Article 83. doi:10.1186/1472-6920-14-83
55. de Galvão e Brito Medeiros A, Lewis S, McNulty J, White P, Lane S, Mackay S. Emotional Intelligence Development in Radiography Curricula: Results of an International Longitudinal Study. J Med Imaging Radiat Sci. 2017;48: 282–287. doi:10.1016/j.jmir.2017.01.001
56. Direktor C, Simsek AH, Serin NB. Negative Automatic Thoughts, Emotional Intelligence and Demographical Different Variables Affecting University Students. Coll Stud J. 2017;51: 391–397.
57. Doherty EM, Cronin PA, Offiah G. Emotional intelligence assessment in a graduate entry medical school curriculum. BMC Med Educ. 2013;13: Article 38. doi:10.1186/1472-6920-13-38
58. Domínguez-Lara S, Sánchez-Carreño K. Uso de estrategias cognitivas de regulación emocional ante la desaprobación de un examen: el rol de la autoeficacia académica en estudiantes universitarios. Psychol Av la Discip. 2017;11: 99–112. doi:10.21500/19002386.2716
59. Dooley D, East L, Nagle C. Emotional intelligence: a qualitative study of student nurses’ and midwives’ theoretical and clinical experience. Contemp Nurse. 2019;55: 341–350. doi:10.1080/10376178.2019.1661784
60. Dubert CJ, Schumacher AM, Locker  Jr. L, Gutierrez AP, Barnes VA, Locker LJ, et al. Mindfulness and emotion regulation among nursing students: Investigating the mediation effect of working memory capacity. Mindfulness (N Y). 2016;7: 1061–1070. doi:10.1007/s12671-016-0544-6
61. Estrada NT, Sosa Rosas MDP. Emotional Intelligence of Undergraduate Nursing Students. Rev Cuid. 2020;11: e993. doi:10.15649/cuidarte.993
62. Eyimaya AO, Ozdemir EA, Tezel A, Duyan V. Evaluation of nursing students’ self-control in Turkey. J Pak Med Assoc. 2020;70: 1013–1017. doi:10.5455/JPMA.38216
63. Farah-Franco S, Singer-Chang G, Deoghare H. Advancing the Measurement of Dental Students’ Professionalism. J Dent Educ. 2017;81: 1338–1344. doi:10.21815/JDE.017.092
64. Fatima A, Ali SK. Relationship of Emotional Intelligence with academic scores and gender in students of Masters in Health Professions Education (MHPE) at a Public Sector University. Pakistan J Med Sci. 2023;39: 1725–1729. doi:10.12669/pjms.39.6.7399
65. Faye A, Kalra G, Swamy R, Shukla A, Subramanyam A, Kamath R. Study of emotional intelligence and empathy in medical postgraduates. Indian J Psychiatry. 2011;53: 140–144. doi:10.4103/0019-5545.82541
66. Fayombo GA. Emotional Intelligence and Gender as Predictors of Academic Achievement among Some University Students in Barbados. Int J High Educ. 2012;1: 102–111. Available: https://search.ebscohost.com/login.aspx?direct=true&db=eric&AN=EJ1055403&lang=es&site=ehost-live&scope=site
67. Fernandez R, Salamonson Y, Griffiths R. Emotional intelligence as a predictor of academic performance in first-year accelerated graduate entry nursing students. J Clin Nurs. 2012;21: 3485–3492. doi:10.1111/j.1365-2702.2012.04199.x
68. Fida A, Ghaffar A, Zaman A, Satti AN. Gender Comparison of Emotional Intelligence of University Students. J Educ Educ Dev. 2018;5: 172–188. Available: https://search.ebscohost.com/login.aspx?direct=true&db=eric&AN=EJ1180616&lang=es&site=ehost-live&scope=site
69. García-Martínez I, Augusto-Landa JM, Quijano-López R, León SP. Self-Concept as a Mediator of the Relation Between University Students’ Resilience and Academic Achievement. Front Psychol. 2022;12: Article 747168. doi:10.3389/fpsyg.2021.747168
70. García-Martínez I, Gavín-Chocano Ó, Molero D, León SP. Analysing university students’ life satisfaction through their socioemotional factors. Rev Investig Educ. 2023;41: 107–124. doi:10.6018/rie.496341
71. Gardner AK, Dunkin BJ. Evaluation of validity evidence for personality, emotional intelligence, and situational judgment tests to identify successful residents. JAMA Surg. 2018;153: 409–416. doi:10.1001/jamasurg.2017.5013
72. Gavín-Chocano Ó, García-Martínez I, Pérez-Navío E, Molero D. Resilience as a Mediating Variable between Emotional Intelligence and Optimism-Pessimism among University Students in Spanish Universities. J Furth High Educ. 2023;47: 407–420. doi:10.1080/0309877X.2022.2133994
73. Getahun Abera W. Emotional intelligence and pro-social behavior as predictors of academic achievement among university students. Community Heal Equity Res Policy. 2023;43: 431–441. https://doi.org/10.1177/0272684X211033447
74. Ghajarzadeh M, Mohammadifar M. Emotional intelligence of medical residents of Tehran University of Medical Sciences. Acta Med Iran. 2013;51: 185–188.
75. Giménez-Espert MDC, Maldonado S, Prado-Gascó V. Influence of Emotional Skills on Attitudes towards Communication: Nursing Students vs. Nurses. Int J Environ Res Public Health. 2023;20: Article 4798. doi:10.3390/ijerph20064798
76. Gleason F, Baker SJ, Wood T, Wood L, Hollis RH, Chu DI, et al. Emotional Intelligence and Burnout in Surgical Residents: A 5-Year Study. J Surg Educ. 2020;77: e63–e70. doi:10.1016/j.jsurg.2020.07.044
77. Gorji AMH, Shafizad M, Soleimani A, Darabinia M, Goudarzian AH. Path analysis of self-efficacy, critical thinking skills and emotional intelligence for mental health of medical students. Iran J Psychiatry Behav Sci. 2018;12: e59487. doi:10.5812/ijpbs.59487
78. Grehan PM, Flanagan R, Malgady RG. Successful Graduate Students: The Roles of Personality Traits and Emotional Intelligence. Psychol Sch. 2011;48: 317–331.
79. Guo M, Yin X, Wang C, Nie L, Wang G. Emotional intelligence a academic procrastination among junior college nursing students. J Adv Nurs. 2019;75: 2710–2718. https://dx.doi.org/10.1111/jan.14101
80. Haight RC, Kolar C, Nelson MH, Fierke KK, Sucher BJ, Janke KK. Assessing emotionally intelligent leadership in pharmacy students. Am J Pharm Educ. 2017;81: 29. https://doi.org/10.5688/ajpe81229
81. Hajibabaee F, A Farahani M, Ameri Z, Salehi T, Hosseini F. The relationship between empathy and emotional intelligence among Iranian nursing students. Int J Med Educ. 2018;9: 239–243. doi:10.5116/ijme.5b83.e2a5
82. Hamah-Morad J, Mostafazadeh A, Areshtanab HN, Ebrahimi H, Bostanabad MA, Hosseinzadeh M. The use of virtual social networks and the relationship to emotional intelligence among nursing students. J Psychiatr Nurs. 2021;12: 307–313. doi:10.14744/phd.2021.14237
83. Hasanpour M, Bagheri M, Heidari FG. The relationship between emotional intelligence and critical thinking skills in Iranian nursing students. Med J Islam Repub Iran. 2018;32: Article 40. doi:10.14196/mjiri.32.40
84. Hasegawa Y, Ninomiya K, Fujii K, Sekimoto T. Emotional intelligence score and performance of dental undergraduates. Odontology. 2016;104: 397–401. doi:10.1007/s10266-015-0219-0
85. Helmich E, Bolhuis S, Prins J, Laan R, Koopmans R. Emotional learning of undergraduate medical students in an early nursing attachment in a hospital or nursing home. Med Teach. 2011;33: e593–e601. doi:10.3109/0142159X.2011.610837
86. Ho MY, Van Tongeren DR, You J. The role of self-regulation in forgiveness: A regulatory model of forgiveness. Front Psychol. 2020;11: Article 1084. https://doi.org/10.3389/fpsyg.2020.01084
87. Hollis RH, Theiss LM, Gullick AA, Richman JS, Morris MS, Grams JM, et al. Emotional intelligence in surgery is associated with resident job satisfaction. J Surg Res. 2017;209: 178–183. doi:10.1016/j.jss.2016.10.015
88. Holman MA, Porter SG, Pawlina W, Juskewitch JE, Lachman N. Does emotional intelligence change during medical school gross anatomy course? Correlations with students’ performance and team cohesion. Anat Sci Educ. 2016;9: 143–149. doi:10.1002/ase.1541
89. Huang C, Zhang L, He T, Wu X, Pan Y, Han Z, et al. The role of emotion regulation in predicting emotional engagement mediated by meta-emotion in online learning environments: A two-stage SEM-ANN approach. Educ Psychol. 2023;43: 736–755. https://doi.org/10.1080/01443410.2023.2254524
90. Hulukati W, Djibran MR. Students’ emotional intelligence at the Faculty of Education at the Universitas Negeri Gorontalo. Int J Innov Creat Chang. 2020;13: 337–348. Available: https://www.scopus.com/inward/record.uri?eid=2-s2.0-85087078483&partnerID=40&md5=5903bd62424e2b62e07cf5b622d874b2
91. Humphrey-Murto S, Leddy JJ, Wood TJ, Puddester D, Moineau G. Does emotional intelligence at medical school admission predict future academic performance? Acad Med. 2014;89: 638–643. doi:10.1097/ACM.0000000000000165
92. Irfan M, Saleem U, Sethi MR, Abdullah AS. Do We Need To Care: Emotional Intelligence And Empathy Of Medical And Dental Students. J Ayub Med Coll Abbottabad. 2019;31: 76–81.
93. Johar N, Ehsan N, Khan MA. Association of Emotional Intelligence with Academic Performance of Medical Students. Pakistan Armed Forces Med J. 2019;69: 455–459. Available: https://www.scopus.com/inward/record.uri?eid=2-s2.0-85083199224&partnerID=40&md5=e3955cc3ba109ff80dc8a87d7dca8f77
94. Joshi B, Emmanuel FJ, Vageriya V. Descriptive study on emotional intelligence amidst nursing undergraduates of choosed nursing institute. Indian J Forensic Med Toxicol. 2020;14: 97–101. doi:10.37506/ijfmt.v14i4.11449
95. Kafetsios K, Maridaki-Kassotaki A, Zammuner VL, Zampetakis LA, Vouzas F. Emotional intelligence abilities and traits in different career paths. J Career Assess. 2009;17: 367–383. doi:10.1177/1069072709334233
96. Karagözoğlu S, Kahve E, Koç O, Adamişoğlu D. Self esteem and assertiveness of final year Turkish university students. Nurse Educ Today. 2008;28: 641–649. doi:10.1016/j.nedt.2007.09.010
97. Kasam L, MacHarapu R, Gade V, Mallepalli P, Babu R, Manjula S. Emotional intelligence among medical undergraduates, interns, postgraduates, and medical practitioners. Arch Ment Heal. 2020;21: 12–15. doi:10.4103/AMH.AMH_16_19
98. Kasemy ZA, Kabbash I, Desouky D, El‑Raouf SA, Aloshari S, Sheikh GE. Perception of educational environment with an assessment of motivational learning strategies and emotional intelligence as factors affecting medical students’ academic achievement. J Educ Health Promot. 2022;11: 303. doi:10.4103/jehp.jehp_1772_21
99. Katrushova L, Yalanska S, Rudenko L, Katrushov O. Peculiarities of the process of psychological adaptation of foreign students of ukrainian higher education institutions of medical profile, role of emotional intelligence in the socialization process. Wiad Lek (Warsaw, Pol 1960). 2019;72: 1930–1934.
100. Kaya H, Şenyuva E, Bodur G. The relationship between critical thinking and emotional intelligence in nursing students: A longitudinal study. Nurse Educ Today. 2018;68: 26–32. doi:10.1016/j.nedt.2018.05.024
101. Khan H, Gupta R, Mishra S. An assessment and correlation of emotional Intelligence and mental health of medical students. Indian J Community Heal. 2022;34: 352–356. doi:10.47203/IJCH.2022.v34i03.006
102. Kilpatrick CC, Doyle PD, Reichman EF, Chohan L, Uthman MO, Orejuela FJ. Emotional intelligence and selection to administrative chief residency. Acad Psychiatry. 2012;36: 388–390. doi:10.1176/appi.ap.10100151
103. Kim J. Factors influencing nursing students’ empathy. Korean J Med Educ. 2018;30: 229–236. doi:10.3946/kjme.2018.97
104. Kim MS, Sohn SK. Emotional intelligence, problem solving ability, self efficacy, and clinical performance among nursing students: A structural equation model. Korean J Adult Nurs. 2019;31: 380–388. doi:10.7475/kjan.2019.31.4.380
105. Kim S-H. The Mediating Effect of Self-Regulated Learning on the Relationships Among Emotional Intelligence, Collaboration, and Clinical Performance in Korean Nursing Students. J Nurs Res. 2022;30: e212. doi:10.1097/jnr.0000000000000494
106. Kim SHS-H, Shin S. Social–emotional competence and academic achievement of nursing students: A canonical correlation analysis. Int J Environ Res Public Health. 2021;18: 1752. doi:10.3390/ijerph18041752
107. Kukulu K, Korukcu O, Ozdemir Y, Bezci A, Calik C. Self-confidence, gender and academic achievement of undergraduate nursing students. J Psychiatr Ment Health Nurs. 2013;20: 330–335. doi:10.1111/j.1365-2850.2012.01924.x
108. Kumar A, Puranik MP, Sowmya KR. Association Between Dental Students’ Emotional Intelligence and Academic Performance: A Study at Six Dental Colleges in India. J Dent Educ. 2016;80: 526–532.
109. Kurian EB, Desai VS, Turner NS, Grawe BM, Kelly AM, Krych AJ, et al. Is Grit the New Fit?-Assessing Non-Cognitive Variables in Orthopedic Surgery Trainees. J Surg Educ. 2019;76: 924–930. doi:10.1016/j.jsurg.2019.01.010
110. Lalonde M, McGillis Hall L. Preceptor characteristics and the socialization outcomes of new graduate nurses during a preceptorship programme. Nurs Open. 2016;4: 24–31. doi:10.1002/nop2.58
111. Larin HM, Benson G, Martin L, Wessel J, Williams R, Ploeg J. Examining change in emotional-social intelligence, caring, and leadership in health professions students. J Allied Health. 2011;40: 96–102.
112. Lawson HJ, Yigah M, Yamson P. Emotional intelligence in medical students at the university of Ghana medical school, Accra, Ghana. Ghana Med J. 2021;55: 52–59. doi:10.4314/GMJ.V55I1.8
113. Lazurenko O, Smila N. Emotional intelligence of future physicians who tend to hide professional mistakes. New Educ Rev. 2021;64: 283–291. doi:10.15804/tner.2021.64.2.23
114. Li Y, Li K, Wei W, Dong J, Wang C, Fu Y, et al. Critical thinking, emotional intelligence and conflict management styles of medical students: A cross-sectional study. Think Ski Creat. 2021;40: 100799. doi:10.1016/j.tsc.2021.100799
115. Liébana-Presa C, Fernández-Martínez E, Morán Astorga C. Relación entre la inteligencia emocional y el burnout en estudiantes de enfermería. Psychol Soc Educ. 2017;9: 335–345. doi:10.25115/psye.v9i3.856
116. Lim G, Gardner AK. Emotional Intelligence and Delivering Bad News: The Jury is Still Out. J Surg Educ. 2019;76: 779–784. doi:10.1016/j.jsurg.2018.09.017
117. Limonero JT, Tomás-Sábado J, Fernández-Castro J, Gómez-Romero MJ, Ardilla-Herrero A. Estrategias de afrontamiento resilientes y regulación emocional: Predictores de satisfacción con la vida. Behav Psychol. 2012;20: 183–196. Available: https://www.scopus.com/inward/record.uri?eid=2-s2.0-84861515975&partnerID=40&md5=b4378ca141d818f6973ae73fee66ad6a
118. Lin DT, Kannappan A, Lau JN. The assessment of emotional intelligence among candidates interviewing for general surgery residency. J Surg Educ. 2013;70: 514–521. doi:10.1016/j.jsurg.2013.03.010
119. Lindeman B, Petrusa E, McKinley S, Hashimoto DA, Gee D, Smink DS, et al. Association of Burnout With Emotional Intelligence and Personality in Surgical Residents: Can We Predict Who Is Most at Risk? J Surg Educ. 2017;74: e22–e30. doi:10.1016/j.jsurg.2017.11.001
120. López-Núñez MI, Rubio-Valdehita S, Díaz-Ramiro EM. The role of individual variables as antecedents of entrepreneurship processes: Emotional intelligence and self-efficacy. Front Psychol. 2022;13: Article 978313. doi:10.3389/fpsyg.2022.978313
121. Mackay S, White P, Mcnulty JP, Lane S, Lewis SJ. A benchmarking and comparative analysis of emotional intelligence in student and qualified radiographers: An international study. J Med Radiat Sci. 2015;62: 246–252. doi:10.1002/jmrs.130
122. Mamcarz I, Torres K, Mamcarz P, Jurek K, Torres A, Szast K. The role of emotional intelligence in attitudes towards elderly patients – comparative study of medical students from rural and urban areas. Ann Agric Environ Med. 2020;27: 134–138. doi:10.26444/aaem/110770
123. Martin Sanz N, Rodrigo IG, Izquierdo García C, Ajenjo Pastrana P. Exploring Academic Performance: Looking beyond Numerical Grades. Univers J Educ Res. 2017;5: 1105–1112. Available: https://search.ebscohost.com/login.aspx?direct=true&db=eric&AN=EJ1147798&lang=es&site=ehost-live&scope=site
124. Martínez-Rodríguez A, Ferreira C. Relationship between academic achievement and emotional intelligence in undergraduate and Master’s Degree students at the University of León. Rev Complut Educ. 2023;34: 795–807. doi:10.5209/rced.80128
125. McGuire C. Relationship Between Resilience, Emotional Intelligence, and Age. Radiol Technol. 2023;95: 8–16. Available: https://www.scopus.com/inward/record.uri?eid=2-s2.0-85171355844&partnerID=40&md5=afe1d881759acffa890427b3cd7877d7
126. Meyer HM. Understanding emotional intelligence and its relationship to clinical reasoning in senior nursing students: A mixed methods study. J Prof Nurs. 2023;46: 187–196. doi:10.1016/j.profnurs.2023.03.010
127. Millán-Franco M, Orgambídez-Ramos A, Domínguez de la Rosa L, Martínez-Martínez SL. La competencia emocional como predictora de la felicidad en trabajadores sociales TT - Emotional competence as a predictor of happiness in social workers. Interdiscip Rev Psicol y Ciencias Afines. 2021;38: 259–274. https://doi.org/10.16888/interd.2021.38.2.17
128. Mintle LS, Greer CF, Russo LE. Longitudinal assessment of medical student emotional intelligence over preclinical training. J Am Osteopath Assoc. 2019;119: 236–242. doi:10.7556/jaoa.2019.039
129. Moghadari-Koosha M, Moghadasi-Amiri M, Cheraghi F, Mozafari H, Imani B, Zandieh M. Self-efficacy, self-regulated learning, and motivation as factors influencing academic achievement among paramedical students: A correlation study. J Allied Health. 2020;49: e145–e152. Available: https://www.proquest.com/scholarly-journals/self-efficacy-regulated-learning-motivation-as/docview/2585456334/se-2?accountid=14777
130. Mohan M, Lin KH, Parolia A, Pau A. Does Emotional Intelligence of Dental Undergraduates Influence Their Patient Satisfaction? Int J Dent. 2021; Article 4573459. doi:10.1155/2021/4573459
131. Montasem A, Brown SL, Harris R. Do core self-evaluations and trait emotional intelligence predict subjective well-being in dental students? J Appl Soc Psychol. 2013;43: 1097–1103. doi:10.1111/jasp.12074
132. Moore A, Canaway R, O’Brien KA. Chinese medicine students’ preparedness for clinical practice: an Australian survey. J Altern Complement Med. 2010;16: 733–743. doi:10.1089/acm.2009.0244
133. Naeem N, van der Vleuten C, Muijtjens AMM, Violato C, Ali SM, Al-Faris EA, et al. Correlates of emotional intelligence: Results from a multi-institutional study among undergraduate medical students. Med Teach. 2014;36: S30–S35. https://dx.doi.org/10.3109/0142159X.2014.886008
134. Niazi A, Qayyum M, Ikram Z, Sher S, Sethi MR, Irfan M. Emotional intelligence and self-perception of medical and dental students of Peshawar-Pakistan. J Postgrad Med Inst. 2019;33: 324–330.
135. Nöthling AC, Khoza TE, Sibiya MN. Benchmarking of Emotional Intelligence in radiography students within KwaZulu- Natal, South Africa. Radiography. 2022;28: 487–491. doi:10.1016/j.radi.2021.10.017
136. Ortiz-Acosta R, Beltrán-Jiménez BE. Perceived emotional intelligence and burnout in undergraduate medicine interns. Educ Medica. 2011;14: 49–55. Available: https://www.scopus.com/inward/record.uri?eid=2-s2.0-79955935305&partnerID=40&md5=6ff154c3c53df815a437069c733b35df
137. Ortiz-Bonnin S, Blahopoulou J, Montañez-Juan MI, García-Buades ME. Team Emotional Intelligence Buffers the Impact of Negative Emotions on Satisfaction with the Team: A Multilevel Study. High Educ Res Dev. 2023;42: 1467–1481. doi:10.1080/07294360.2022.2152781
138. Parmentier M, Pirsoul T, Nils F. Career Adaptability Profiles and Their Relations with Emotional and Decision-Making Correlates among Belgian Undergraduate Students. J Career Dev. 2022;49: 934–950. doi:10.1177/08948453211005553
139. Partido BB, Stefanik D, Forsythe A. Association between emotional intelligence and professionalism among dental hygiene students. J Dent Educ. 2020;84: 1341–1347. doi:10.1002/jdd.12327
140. Partido BB, Stefanik D, Rashid W. Relationship between emotional intelligence and professionalism among second-year dental students. J Dent Educ. 2021;85: 411–417. doi:10.1002/jdd.12467
141. Pau A, Sabri BA. Relationship between emotional intelligence and job satisfaction in newly qualified Malaysian dentists. Asia-Pacific J Public Heal. 2015;27: NP1733–NP1741. doi:10.1177/1010539512449855
142. Pavlova EM, Kornilova T V. Creativity and tolerance for uncertainty predict the engagement of emotional intelligence in personal decision making. Psychol Russ State Art. 2013;6: 34–46. https://dx.doi.org/10.11621/pir.2013.0403
143. Pawełczyk A, Kotlicka-Antczak M, Śmigielski J, Pawełczyk T, Rabe-Jabłońska J. Emotional intelligence and medical specialty preference - Findings from the empirical study. Psychiatr i Psychol Klin. 2012;12: 96–101.
144. Pence PL. Predictors of retention among undergraduate students attending associate-degree nursing programs in Illinois. Teach Learn Nurs. 2011;6: 131–138. doi:10.1016/j.teln.2011.01.004
145. Pendyala SK, Kondreddy K, Hau LL, Phing LC, Ying LP, Lim L. Emotional intelligence among undergraduate dental students and its relationship with academic and clinical performance. Int J Dent Oral Sci. 2021;8: 2140–2145. https://dx.doi.org/10.19070/2377-8075-21000423
146. Percy DB, Streith L, Wong H, Ball CG, Widder S, Hameed M. Mental toughness in surgeons: Is there room for improvement? Can J Surg. 2019;62: 482–487. doi:10.1503/cjs.010818
147. Pérez-Bonet G, Velado Guillén LÁ, García-Domingo B, Sánchez-Fernández ML. Emotional intelligence and early maladaptive schemas in future educators: Expanding borders. Rev Electron Interuniv Form del Profr. 2021;24: 133–147. doi:10.6018/REIFOP.435821
148. Pérez-Fuentes M, Molero-Jurado M, Simón-Márquez M, Barragán-Martín AB, Martos-Martínez Á, Ruiz-Oropesa NF, et al. Academic engagement and emotional intelligence in health sciences students. Rev Psicol y Educ. 2020;15: 77–86. Available: https://www.proquest.com/scholarly-journals/engagement-académico-e-inteligencia-emocional-en/docview/2504837884/se-2?accountid=14777
149. Pirsoul T, Parmentier M, Nils F. Emotional Intelligence Profiles and Job Search Correlates in the Context of the School-to-Work Transition. J Career Dev. 2023;50: 1038–1057. doi:10.1177/08948453221141445
150. Polonio-López B, Triviño-Juárez J-M, Corregidor-Sánchez AI, Toledano-González A, Rodríguez-Martínez MC, Cantero-Garlito P, et al. Improving self-perceived emotional intelligence in Occupational Therapy students through practical training. Front Psychol. 2019;10: Article 920. doi:10.3389/fpsyg.2019.00920
151. Pope D, Roper C, Qualter P. The Influence of Emotional Intelligence on Academic Progress and Achievement in UK University Students. Assess Eval High Educ. 2012;37: 907–918.
152. Pradhan RK, Jandu K. Evaluating the impact of conscientiousness on flourishing in Indian higher education context: Mediating role of emotional intelligence. Psychol Stud (Mysore). 2023;68: 223–235. https://doi.org/10.1007/s12646-022-00712-4
153. Prieto De Rincón D, Inciarte-Mundo J, Rincón-Prieto C, Bonilla E. The emotional coefficient in medicine students. Rev Chil Neuropsiquiatr. 2008;46: 10–15. Available: https://www.scopus.com/inward/record.uri?eid=2-s2.0-51849155499&partnerID=40&md5=efda8f7662a8082668140d9419cdf228
154. Purnamaningsih EH. Personality and emotion regulation strategies. Int J Psychol Res. 2017;10: 53–60. doi:10.21500/20112084.2040
155. Rankin B. Emotional intelligence: Enhancing values-based practice and compassionate care in nursing. J Adv Nurs. 2013;69: 2717–2725. doi:10.1111/jan.12161
156. Razavi SM, Omid A, Rezaei H, Khalesi S. The Correlation among Emotional Intelligence, Motivated Strategies for Learning and the Academic Performance of Dental Students in Isfahan University of Medical Sciences during 2017-2018. J Med Educ Dev. 2020;13: 81–88. doi:10.29252/edcj.13.37.85
157. Rentzios C, Kamtsios S, Karagiannopoulou E. The Mediating Role of Implicit and Explicit Emotion Regulation in the Relationship Between Academic Emotions and Approaches to Learning. J Nerv Ment Dis. 2019;207: 683–692. doi:10.1097/NMD.0000000000001027
158. Rodríguez RPS, Jiménez M de la VM, de la Villa Moral Jiménez M. Empatía, inteligencia emocional y autoestima en estudiantes universitarios de carreras sanitarias. Electron J Res Educ Psychol. 2022;20: 311–334. doi:10.25115/ejrep.v20i57.5083
159. Roos TC, Niehaus DJH, Leppänen JM, Ras J, Cloete KJ, Jordaan E, et al. Facial affect recognition and exit examination performance in medical students: A prospective exploratory study. BMC Med Educ. 2014;14: 245. doi:10.1186/s12909-014-0245-6
160. Sa B, Baboolal N, Williams S, Ramsewak S. Exploring emotional intelligence in a Caribbean medical school. West Indian Med J. 2014;63: 159–166. doi:10.7727/wimj.2013.124
161. Salvador-Ferrer CM. Achievement Motivation and Goals in Life: The Mediating Role of Emotional Intelligence. Electron J Res Educ Psychol. 2021;19: 1–18. Available: https://search.ebscohost.com/login.aspx?direct=true&db=eric&AN=EJ1293281&lang=es&site=ehost-live&scope=site
162. Sánchez Expósito J, Jiménez-Rodríguez D, Díaz Agea JL, Carrillo Izquierdo MD, Leal Costa C. Impact of Socio-Emotional Skills On The Performance of Clinical Nursing Practices. Int J Nurs Educ Scholarsh. 2019;16: Article 20190064. doi:10.1515/ijnes-2019-0064
163. Sanchez-Ruiz MJ, Mavroveli S, Poullis J. Trait emotional intelligence and its links to university performance: An examination. Pers Individ Dif. 2013;54: 658–662. https://dx.doi.org/10.1016/j.paid.2012.11.013
164. Sánchez-Ruiz MJ, Pérez-González JC, Petrides K V. Trait emotional intelligence profiles of students from different university faculties. Aust J Psychol. 2010;62: 51–57. https://dx.doi.org/10.1080/00049530903312907
165. Sarikaya O, Yegen B. Multisource Assessment in Conjunction with Emotional Intelligence Can Be Used for Monitoring the Development of Residents’ Professional Competencies. Med Sci Educ. 2017;27: 527–534. doi:10.1007/s40670-017-0430-4
166. Sasanpour M, Khodabakhshi K, Nooryan K. The relationship between emotional intelligence, happiness and mental health in students of medical sciences of Isfahan university. Int J Collab Res Intern Med Public Heal. 2012;4: 1614–1620. Available: https://www.scopus.com/inward/record.uri?eid=2-s2.0-84867127199&partnerID=40&md5=b7b4df21415c942fff43e31623a65989
167. Schofield R, Allan M, Jewiss T, Hunter A, Sinclair N, Diamond A, et al. Knowing self and caring through service learning. Int J Nurs Educ Scholarsh. 2013;10: 267–274. doi:10.1515/ijnes-2013-0009
168. Shabbani MB, Latifi GR, Javaheri R, Mazlum M. Undergraduate students’ emotional intelligence and their perceptions of learner autonomy: Interface between social science and English language students. Cogent Educ. 2020;7: 1850–194. doi:10.1080/2331186X.2020.1850194
169. Sheikhbardsiri H, Sheikhasadi H, Mahani S, Mohamadi M. Emotional intelligence and learning strategies of postgraduate students at Kerman University of Medical Sciences in the southeast of Iran. J Educ Health Promot. 2020;9: 66. doi:10.4103/jehp.jehp_544_19
170. Silva CG, Gordo S, Rodrigues AC, Henriques C, Rosa M. Exploring the Relationship between Socioemotional Skills and Decision-Making Styles in Health Students. IAFOR J Educ. 2021;9: 49–65. Available: https://search.ebscohost.com/login.aspx?direct=true&db=eric&AN=EJ1318683&lang=es&site=ehost-live&scope=site
171. Singer-Chang G, Dong F, Seffinger M, Nevins N, Blumer J, Musharbash H, et al. Empathy in medicine self and other in medical education: Initial emotional intelligence trend analysis widens the lens around empathy and burnout. J Am Osteopath Assoc. 2020;120: 388–394. doi:10.7556/jaoa.2020.069
172. Smith MJ, Wilson J, George DL, Laster K, Filippo C, Spies A. Emotional intelligence scores among three cohorts of pharmacy students before and after completing the University of Oklahoma College of Pharmacy’s Leadership Degree Option Program. Curr Pharm Teach Learn. 2018;10: 911–917. doi:10.1016/j.cptl.2018.04.001
173. Snowden A, Stenhouse R, Duers L, Marshall S, Carver F, Brown N, et al. The relationship between emotional intelligence, previous caring experience and successful completion of a pre-registration nursing/midwifery degree. J Adv Nurs. 2018;74: 433–442. doi:10.1111/jan.13455
174. Snowden A, Stenhouse R, Young J, Carver H, Carver F, Brown N. The relationship between emotional intelligence, previous caring experience and mindfulness in student nurses and midwives: A cross sectional analysis. Nurse Educ Today. 2015;35: 152–158. doi:10.1016/j.nedt.2014.09.004
175. Southward MW, Cheavens JS. More (of the right strategies) is better: Disaggregating the naturalistic between- and within-person structure and effects of emotion regulation strategies. Cogn Emot. 2020;34: 1729–1736. https://doi.org/10.1080/02699931.2020.1797637
176. Stenhouse R, Snowden A, Young J, Carver F, Carver H, Brown N. Do emotional intelligence and previous caring experience influence student nurse performance? A comparative analysis. Nurse Educ Today. 2016;43: 1–9. doi:10.1016/j.nedt.2016.04.015
177. Štiglic G, Cilar L, Novak Ž, Vrbnjak D, Stenhouse R, Snowden A, et al. Emotional intelligence among nursing students: Findings from a cross-sectional study. Nurse Educ Today. 2018;66: 33–38. doi:10.1016/j.nedt.2018.03.028
178. Stratton TD, Elam CL, Murphy-Spencer AE, Quinlivan SL. Emotional intelligence and clinical skills: Preliminary results from a comprehensive clinical performance examination. Acad Med. 2005;80: S34–S37. doi:10.1097/00001888-200510001-00012
179. Strickland HP, Cheshire MH. Exploring the Correlation Between Nontraditional Variables and Student Success: A Longitudinal Study. J Nurs Educ. 2017;56: 351–355. doi:10.3928/01484834-20170518-06
180. Talarico JF, Metro DG, Patel RM, Carney P, Wetmore AL. Emotional intelligence and its correlation to performance as a resident: a preliminary study. J Clin Anesth. 2008;20: 84–89. doi:10.1016/j.jclinane.2007.12.008
181. Talarico JF, Varon AJ, Banks SE, Berger JS, Pivalizza EG, Medina-Rivera G, et al. Emotional intelligence and the relationship to resident performance: A multi-institutional study. J Clin Anesth. 2013;25: 181–187. doi:10.1016/j.jclinane.2012.08.002
182. Talman K, Hupli M, Rankin R, Engblom J, Haavisto E. Emotional intelligence of nursing applicants and factors related to it: A cross-sectional study. Nurse Educ Today. 2020;85: 104271. doi:10.1016/j.nedt.2019.104271
183. Taneja N, Gupta S, Chellaiyan V, Awasthi A, Sachdeva S. Personality traits as a predictor of emotional intelligence among medical students. J Educ Health Promot. 2020;9: Article 354. doi:10.4103/jehp.jehp_678_19
184. Tariq SS, Tariq SS, Atta K, Rehman R, Ali Z. Emotional Intelligence: A predictor of undergraduate student’s academic achievement in altered living conditions. J Pak Med Assoc. 2020;70: 2398–2402. doi:10.47391/JPMA.429
185. Treat R, Hueston WJ, Fritz J, Prunuske A, Hanke CJ. Medical Student Burnout as Impacted by Trait Emotional Intelligence – Moderated by Three-Year and Four-Year Medical Degree Programs and Gender. Wis Med J. 2021;120: 188–194. Available: https://www.scopus.com/inward/record.uri?eid=2-s2.0-85125616957&partnerID=40&md5=5237c7e864a082592234e31f443306d2
186. Turan N, Özdemir Aydın G, Özsaban A, Kaya H, Aksel G, Yılmaz A, et al. Intuition and emotional intelligence: A study in nursing students. Cogent Psychol. 2019;6: 1633077. doi:10.1080/23311908.2019.1633077
187. Tyszkiewicz-Bandur M, Walkiewicz M, Tartas M, Bankiewicz-Nakielska J. Emotional intelligence, attachment styles and medical education. Fam Med Prim Care Rev. 2017;19: 404–407. doi:10.5114/fmpcr.2017.70127
188. Ulupınar S, Şenyuva E, Küçük Yüceyurt N. Does participation of nursing students in social activities affect their social emotional learning skills? Nurse Educ Today. 2019;76: 78–84. doi:10.1016/j.nedt.2019.01.031
189. Van Eckert S, Gaidys U, Martin CR. Self-esteem among German nurses: does academic education make a difference? J Psychiatr Ment Health Nurs. 2012;19: 903–910. doi:10.1111/j.1365-2850.2011.01862.x
190. Vasefi A, Dehghani M, Mirzaaghapoor M. Emotional intelligence of medical students of Shiraz University of Medical Sciences cross sectional study. Ann Med Surg. 2018;32: 26–31. doi:10.1016/j.amsu.2018.07.005
191. Vilca-Pareja V, Luque Ruiz de Somocurcio A, Delgado-Morales R, Medina Zeballos L. Emotional Intelligence, Resilience, and Self-Esteem as Predictors of Satisfaction with Life in University Students. Int J Environ Res Public Health. 2022;19: Article 16548. doi:10.3390/ijerph192416548
192. Volberding JL, Baghurst T, Brown TC. Emotional intelligence levels of undergraduate kinesiology students: Brief report. N Am J Psychol. 2015;17: 37–44.
193. Wang Y, Zhang Y, Liu M, Zhou L, Zhang J, Tao H, et al. Research on the formation of humanistic care ability in nursing students: A structural equation approach. Nurse Educ Today. 2020;86: 104315. doi:10.1016/j.nedt.2019.104315
194. Weise C, Aguayo-González M, Castelló M. Significant events and the role of emotion along doctoral researcher personal trajectories. Educ Res. 2020;62: 304–323. https://doi.org/10.1080/00131881.2020.1794924
195. Wijekoon CN, Amaratunge H, De Silva Y, Senanayake S, Jayawardane P, Senarath U. Emotional intelligence and academic performance of medical undergraduates: A cross-sectional study in a selected university in Sri Lanka. BMC Med Educ. 2017;17: Article 176. doi:10.1186/s12909-017-1018-9
196. Wood TJ, Humphrey-Murto S, Moineau G, Forgie M, Puddester D, Leddy J. Does emotional intelligence at medical school admission predict licensing examination performance? Can Med Educ J 2020. 2020;11: e35–e45. doi:10.1097/ACM.0000000000000165
197. Xiao H, Double KS, Walker SA, Kunst H, MacCann C. Emotionally Intelligent People Use More High-Engagement and Less Low-Engagement Processes to Regulate Others’ Emotions. J Intell. 2022;10: Article 76. doi:10.3390/jintelligence10040076
198. Yang Y. The association of daily stressors and daily emotions to daily optimism: The role of emotion differentiation. J Individ Differ. 2022;43: 216–227. https://doi.org/10.1027/1614-0001/a000375
199. Yasmeen R, Shah AA, Naseer S, Syeda ZF. Teacher-class relationship and emotional intelligence in the academic output and generic competence of higher education students. PLoS One. 2023;18: e0292120. doi:10.1371/journal.pone.0292120
200. Yen W, Hovey R, Hodwitz K, Zhang S. An exploration of the relationship between emotional intelligence (EI) and the Multiple Mini-Interview (MMI). Adv Heal Sci Educ. 2011;16: 59–67. doi:10.1007/s10459-010-9241-8
201. Zhou X, Sun X, Wang Z, Jiang T. Association between conscientiousness and team emotional intelligence: A moderated mediation model. Med (United States). 2022;101: E31001. doi:10.1097/MD.0000000000031001
