# Supplementary material for: Strategies to assess and promote the socio-emotional competencies of university students in the socio-educational and healthcare fields: A scoping review
Source: PLoS One. 2025 May 22;20(5):e0324531. doi: 10.1371/journal.pone.0324531 (PMC12097715; doi:10.1371/journal.pone.0324531)
Supplement: Attachment — Submitted filename: Response to reviewers.docx [file pone.0324531.s017.docx]

Authors’ response. We would like to thank you and the reviewers for providing valuable feedback on how to improve the manuscript. We greatly appreciate your consideration and the reviewers’ comments, and we have implemented the proposed changes as suggested.

Below you will find our point-by-point responses to each issue raised by the reviewers. Every change listed in this letter is also present in the revised manuscript. The changes made in the manuscript have been indicated in purple so that the editor and reviewers can easily locate them.

We look forward to hearing from you.

Yours sincerely.

**Editor:**

1. **First, you should expand the discussion on the practical implications of Social-Emotional Competencies (SECs), focusing on how the findings can inform curriculum design in healthcare and socio-educational fields. Additionally, it explores how SEC training can be institutionalized within higher education policies to ensure its integration into academic frameworks.**

Authors’ response. Thanks for your note. In lines 581 to 597, we discuss how SECs can be integrated into curricula in the socio-educational and healthcare fields. Based on frameworks such as the Bologna Declaration (1999) and the European Association for Quality Assurance in Higher Education (2015), we highlight the importance of combining academic knowledge with transferable skills to enhance students' personal and professional development. Additionally, we propose a specific approach for their curricular incorporation.

2. **Next, consider enhancing the comparative analysis of SEC measurement tools by including a table outlining key assessment tools' psychometric properties such as validity and reliability. It would also be beneficial to clarify which tools are most commonly used and why they are popular.**

Authors’ response. We appreciate your suggestion. A column has been added to Table 1 detailing the number of studies that report adequate validity and reliability indices. Additionally, in the Datasets 1-3 you can find the specific indices for each instrument from all studies that report them. Lastly, in lines 489 to 492 we have provided an explanation of why the mentioned measures are among the most commonly used.

3. **Addressing the socio-educational representation gap is another critical area to strengthen. Develop a more structured argument explaining why the socio-educational field has lagged in SEC research and propose potential policy recommendations or funding priorities that could help bridge this gap.**

Authors’ response. We agree. In lines 454 to 462, we have already provided an explanation of why this gap may exist in SEC research within the socio-educational field. However, we have expanded the argument further in lines 462 to 475, offering a more structured discussion and a specific example of the need for SECs in the socio-educational field.

4. **Furthermore, in the section on intervention studies, a systematic review of best practices could be improved by creating a matrix that categorizes interventions based on their effectiveness, target competencies, and methodologies employed.**

Authors’ response. Thank you for your suggestion. Upon considering your recommendation, we realised that the studies we examined do not provide detailed strategies for each SEC, which limits the possibility of constructing such a matrix. The aim of this scoping review is to identify and describe the intervention programs developed to promote SECs at the university level, focusing on their theoretical frameworks, target SECs, and outcomes. Given that our primary goal is to map the landscape of SEC interventions, rather than to establish a best practices framework, the creation of such a matrix falls beyond the scope of this review. However, we do present findings on the effectiveness and efficiency of the interventions in lines 419 to 437, and specific data on these aspects can be found in column V of Dataset 3.

5. **Lastly, it is important to strengthen the methodology section by providing greater transparency in data extraction and synthesis processes to enhance reproducibility. Additionally,** **explicitly acknowledging potential biases in literature selection—such as language and publication biases—will strengthen the manuscript's credibility.**

Authors’ response. Thank you for pointing this out. On the one hand, as described in lines 173 to 174, data extraction was performed according to a predefined coding protocol. Additionally, to enhance transparency and reproducibility, we have added a supplementary table (S3 Table) that provides a detailed description of the variables and their corresponding definitions. On the other hand, in lines 657 to 661, we explicitly acknowledge the potential biases in literature selection.

**Reviewer 1:**

1. **Clarification of Theoretical Frameworks. While the manuscript mentions various theoretical models of SECs, there is a need for a clearer synthesis of these frameworks to guide readers. A concise table summarizing the key models and their respective SECs dimensions would be helpful.**

Authors’ response. We appreciate your valuable suggestion. To provide clearer guidance to readers, we have included Table S1, which summarizes the key theoretical models and their respective SECs.

2. **Greater Emphasis on the Socio-Educational Field. The review acknowledges the dominance of healthcare studies in SECs research, yet it would benefit from a more detailed discussion on why this gap exists and how future research can better address it. More specific examples of the role SECs play in socio-educational professions would strengthen this section**.

Authors’ response. We have already addressed this valuable contribution as previous mentioned in Editor Comment 3. In lines 454 to 462, we have already provided an explanation of why this gap may exist in SEC research within the socio-educational field. However, we have expanded the argument further in lines 462 to 475, offering a more structured discussion and a specific example of the need for SECs in the socio-educational field.

3. **Intervention Studies: Standardization and Longitudinal Analysis
   The paper highlights the heterogeneity of SECs intervention studies but could provide a more structured analysis of best practices. A comparison table outlining the most effective intervention strategies and their outcomes would be beneficial.**

Authors’ response. Thank you for your suggestion, as previous mentioned in Editor Comment 4, upon considering your recommendation, we realised that the studies we examined do not provide detailed strategies for each SEC, which limits the possibility of constructing such a matrix. The aim of this scoping review is to identify and describe the intervention programs developed to promote SECs at the university level, focusing on their theoretical frameworks, target SECs, and outcomes. Given that our primary goal is to map the landscape of SEC interventions, rather than to establish a best practices framework, the creation of such a matrix falls beyond the scope of this review. However, we do present findings on the effectiveness and efficiency of the interventions in lines 419 to 437, and specific data on these aspects can be found in column V of Dataset 3.

4. **The need for longitudinal studies to assess the long-term impact of SECs training is briefly mentioned but should be emphasized further.**

Authors’ response. According to your comment, the importance of longitudinal studies to assess the long-term impact of SEC training has been further emphasized in lines 640 to 645.

5. **Limitations and Reporting Transparency Some details regarding study selection and data extraction processes could be expanded to further ensure transparency and reproducibility. Given the reliance on published studies, potential publication bias should be acknowledged more explicitly, with suggestions for addressing this issue in future research.**

Authors’ response. Firstly, as previous mentioned in Editor Comment 5, we have added a table (S3 Table) to enhance transparency and reproducibility. This table provides a detailed description of the variables and their corresponding definitions. Secondly, in lines 608 to 629, we explicitly address potential publication bias by citing two reference guidelines for tackling this issue and proposing specific elements that need to be reported in future research to mitigate this bias.

6. **Language and Readability. The manuscript is well-written but could benefit from minor refinements to enhance clarity and readability. Some sections are dense with technical details; breaking up long paragraphs or simplifying certain explanations could improve reader engagement.**

Authors’ response. Thank you for your valuable feedback. We have taken your suggestion into account and made several adjustments throughout the manuscript. Some of the modifications can be seen in the Introduction (lines 67 to 71; 84 to 87), Methodology (lines 183 to 184), Results (lines 234 to 235; 315 to 319) or Discussion (lines 677 to 680).

**Reviewer 2:**

**1. On page 13, lines 273–280, the study notes the heterogeneity of instruments used to assess social and educational competencies. The authors could expand their discussion on the need to develop and adopt standardized measures specifically designed to assess SECs.**

Authors’ response. We appreciate your input on this point. In lines 497 to 498, the discussion has been expanded to address the biases associated with measuring SECs using instruments not specifically designed for this purpose.

**2. On page 20, lines 345–347, it appears that 69% of intervention studies are identified, limiting the replicability of the theoretical framework. The authors could emphasize this as a recommendation in the discussion in line with established frameworks.**

Authors’ response. According to your comment, this point has already been addressed in lines 519 to 522, where we emphasize the importance of establishing a theoretical framework for interventions, as it provides a structured foundation for organizing information, communicating evidence, and guiding actions to achieve expected outcomes.

**3. Page 21, lines 374–389: Many intervention studies lack information on the number, duration, and frequency of sessions. The authors should recommend improved reporting standards for future intervention research and highlight the need for better intervention reporting guidelines.**

Authors’ response. In agreement with your comment, in lines 608 to 629 we have added recommendations for future research to adopt standardized reporting guideline. Moreover, we explicitly outline key aspects of intervention implementation, including integration into curricular activities, scheduling details, instructor expertise, delivery methods, and expected outcomes.

**4. Page 22, Lines 405-416: The variables, such as gender and academic year, are identified as moderators in some intervention studies. The discussion could explore the implications of these findings more thoroughly, particularly how interventions could be tailored based on these factors**.

Authors’ response. We appreciate your suggestion. We have expanded the discussion in lines 560 to 569 to further explore how interventions could be tailored considering gender and academic year as moderating variables. This addition highlights the potential implications of these factors in designing more effective.

**5. In figure 4, ensure that captions are detailed and self-explanatory for standalone readability and would benefit from a brief summary of the categories in their captions.**

Authors’ response. Thank you for pointing this out. The caption for Figure 4 has been revised to include detailed descriptions of each category, ensuring that it is clear and comprehensible on its own.

**6. Maintain consistent use of terms such as “social and emotional competencies” and “emotional intelligence” throughout the manuscript to avoid confusing the reader.**

Authors’ response. Thank you for your observation. We have ensured consistent use of the term *social and emotional competencies* throughout the manuscript. The term *emotional intelligence* only appears when referring to specific theoretical frameworks or measurement tools.

**7. The authors state that they follow the PRISMA-ScR guidelines; it would be nice to include the checklist in the supplementary materials for confirmation.**

Authors’ response. The PRISMA-ScR checklist has been included in the supplementary S1 Checklist, where it can be reviewed for confirmation. Please let us know if any additional details are required.
